# Quantitative analysis of the blood transcriptome of young healthy pigs and its relationship with subsequent disease resilience

**DOI:** 10.1186/s12864-021-07912-8

**Published:** 2021-08-12

**Authors:** Kyu-Sang Lim, Jian Cheng, Austin Putz, Qian Dong, Xuechun Bai, Hamid Beiki, Christopher K. Tuggle, Michael K. Dyck, Pig Gen Canada, Frederic Fortin, John C. S. Harding, Graham S. Plastow, Jack C. M. Dekkers

**Affiliations:** 1 Department of Animal Science, Iowa State University, Ames, Iowa, 50011 USA; 2 Swine Business Unit, Hendrix Genetics, Boxmeer, 5831 CK The Netherlands; 3 Department of Epidemiology and Cancer Control, St. Jude Children’s Research Hospital, Memphis, TN 38105 USA; 4 Department of Agriculture, Food and Nutritional Science, University of Alberta, Edmonton, AB T6G 2R3 Canada; 5 PigGen Canada Research Consortium, Guelph, Ontario N1H4G8 Canada; 6 Centre de Développement du Porc du Québec Inc. (CDPQ), Québec City, QC G1V 4M6 Canada; 7 Department of Large Animal Clinical Sciences, University of Saskatchewan, Saskatoon, SK S7N 5A2 Canada

**Keywords:** Pigs, Disease resilience, Disease challenge, Blood, Transcriptomics

## Abstract

**Background:**

Disease resilience, which is the ability of an animal to maintain performance under disease, is important for pigs in commercial herds, where they are exposed to various pathogens. Our objective was to investigate population-level gene expression profiles in the blood of 912 healthy F1 barrows at ~ 27 days of age for associations with performance and health before and after their exposure to a natural polymicrobial disease challenge at ~ 43 days of age.

**Results:**

Most significant (*q* < 0.20) associations of the level of expression of individual genes in blood of young healthy pigs were identified for concurrent growth rate and subjective health scores prior to the challenge, and for mortality, a combined mortality-treatment trait, and feed conversion rate after the challenge. Gene set enrichment analyses revealed three groups of gene ontology biological process terms that were related to disease resilience: 1) immune and stress response-related terms were enriched among genes whose increased expression was unfavorably associated with both pre- and post-challenge traits, 2) heme-related terms were enriched among genes that had favorable associations with both pre- and post-challenge traits, and 3) terms related to protein localization and viral gene expression were enriched among genes that were associated with reduced performance and health traits after but not before the challenge.

**Conclusions:**

Gene expression profiles in blood from young healthy piglets provide insight into their performance when exposed to disease and other stressors. The expression of genes involved in stress response, heme metabolism, and baseline expression of host genes related to virus propagation were found to be associated with host response to disease.

**Supplementary Information:**

The online version contains supplementary material available at 10.1186/s12864-021-07912-8.

## Background

Disease resilience is a comprehensive concept that integrates resistance and tolerance [1–3], which are sequential shields that protect animals from disease agents. Resistance can be defined as the ability of the host to limit an increase in pathogen level in the host as external pathogen exposure increases. Tolerance can be defined as the ability to limit the impact of an increase in pathogen level in the host on its performance. Disease resilience can be defined as the ability to limit the impact of an increase in external exposure to the pathogen on the host’s performance [4]. In contrast to resistance and tolerance, disease resilience does not require measurement of pathogen load at the individual level, which is very difficult in terms of cost and labor [2, 3]. In addition, with the large number of pathogens for pigs worldwide, measuring resistance and/or tolerance for one pathogen may not predict these measures for another pathogen. Disease resilience does not require determination of pathogen burden and also applies when multiple pathogens are present. Hence, disease resilience represents a very useful concept for the improvement of animal populations that face health challenges, such as in commercial pig production.

RNA-sequencing (RNA-seq) of full length transcripts is a widely used method to quantify gene expression levels in blood samples and has been applied in several studies to investigate host response to important pig pathogens such as porcine reproductive and respiratory syndrome virus [5–7], African swine fever virus [8], foot-and-mouth disease virus [9], and mycoplasma [10]. In each of these studies, pigs were artificially infected with a single dose of the targeted pathogen in order to investige changes in gene expression after infection. In all but one case [7], less than 100 samples were used, reflecting the relatively high cost of full-length RNA-seq. In addition, most studies applied depletion of globin RNAs [5–7, 10] or of rRNA [8] prior to RNA-seq to increase sensitivity. This additional step increases labor. To overcome these limitations, we applied QuantSeq 3’mRNA sequencing (QuantSeq) with Globin-blocker (GB) (QuantSeq, Lexogen, Austria), as described in [11]. To generate QuantSeq libraries, no prior steps for poly(A) enrichment and rRNA depletion are needed because total RNA is used as input and starts with oligodT priming. QuantSeq sequences only the 3’end of transcripts [12] and, in combination with GB, reduces the sequence space needed to adequately explore the transcriptome of blood samples.

Gene expression levels in heterogeneous tissue samples, including whole blood, can be affected by cell composition of the samples, making it difficult to determine whether differences in mRNA read counts for a gene between samples are due to differences in expression of the gene, differences in cell composition, or both [13]. Recently, single-cell RNA sequencing has received much attention due to it’s ability to distinguish heterogeneous gene expression patterns in different cell types that are present in a complex sample, as reviewed by Hwang et al. [14]. Also, deconvolution of gene expression data for a heterogeneous sample into estimates of gene expression levels of individual cell types have been proposed, using the gene expression signatures of 64 immune and stromal cell types [15] and of 29 immune cell types within peripheral blood mononuclear cells [16]. However, comprehensive cell type signatures have not been reported for the pig. Whitney et al. [17] reported associations of gene expression patterns in blood from healthy human donors with relative proportions of specific blood cell subsets, supporting the application of white blood cell (WBC) composition to adjust gene expression levels.

Here, we measured RNA levels in a large set of blood samples collected on young healthy piglets, prior to their exposure to a natural polymicrobial disease challenge, as described in [18]. The resulting data were used to identify genes whose expression in blood in young healthy piglets is associated with concurrent performance and with their performance and resilience following exposure to polymicrobial infectious agents. Expression values were adjusted by mixed linear models with (eWI) or without (eWO) accounting for WBC composition to address the cellular heterogeneity of the blood samples analyzed. The adjusted expression values for a gene were then used for quantitative analysis of associations with concurrent performance and with subsequent disease resilience by fitting gene expression as a covariate for continuous traits or as a response variable for binary traits in trait-specific mixed linear models.

## Results

### Resilience traits under a natural disease challenge

**Fig. 1:**
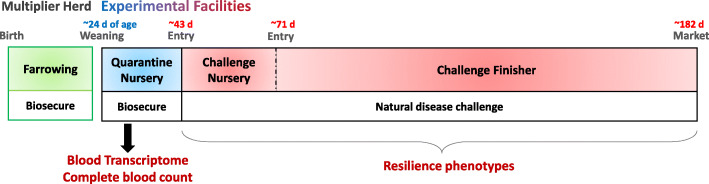
Illustration of the natural disease challenge model

A total of 912 pigs in 15 batches from the natural disease challenge model (NDCM) [18, 19], illustrated in Fig. 1, were used in the current analysis. Population-scale blood transcriptomic data from young healthy pigs were used to determine associations with multiple phenotypes collected before and after exposure to a polymicrobial disease challenge. Blood samples for transcriptome analysis were collected at ~ 27 days of age while the pigs were acclimating in a biosecure quarantine nursery (qNUR). Two weeks later, the pigs were moved to a nearby natural disease challenge nursery and finisher (cNUR and FIN), as described in [18]. Disease resilience and performance traits were evaluated across the nursery and finisher phases, including subjective health scores (HS), health treatment rates (TRT), mortality (MOR), growth rate, feed efficiency, and carcass traits. Records for mortality and treatments were also combined into a new binary trait (MT) that classified animals as died versus survived without treatment, with animals that survived with treatment set to missing. Summary statistics of all phenotypes are provided in Table 1. The number of pigs evaluated differed by trait because of mortality. Mortality rates were similar in the challenge nursery (12%) and finisher (13%), despite the much shorter length of the challenge nursery phase (27 days) than the finisher phase (100 days), reflecting the higher disease pressure in the challenge nursery, where pigs were first exposed to disease. Summary statistics for these 15 batches were similar to the descriptive statistics of traits across 50 batches of the NDCM (3285 pigs) as presented in [19], which included the 15 batches used here.

**Table 1:** Descriptive statistics of the evaluated phenotypes by trait category and growth phase

Category	Phase	Trait abbreviation (units)	# of pigs	Mean	Standard deviation
Subjective health score	Quarantine nursery	qNurHS1	912	4.85	0.36
		qNurHS2	912	4.86	0.35
	Challenge nursery	NurHS	894	4.46	0.69
	Finisher	FinHS	761	4.78	0.49
Treatment rate	Challenge nursery	NurTRT (per 27 days)	903	1.07	1.09
	Finisher	FinTRT (per 100 days)	746	0.28	0.70
	Nursery + finisher	AllTRT (per 180 days)	778	1.26	1.34
Mortality plus treatments	Challenge nursery	NurMT	427	0.51	0.87
	Finisher	FinMT	694	0.31	0.72
	Nursery + finisher	AllMT	482	0.89	1.00
Mortality	Challenge nursery	NurMOR	912	0.12	0.32
	Finisher	FinMOR	803	0.13	0.34
	Nursery + finisher	AllMOR	912	0.24	0.42
Growth rate	Quarantine nursery	qNurADG (kg/day)	912	0.31	0.09
	Challenge nursery	NurADG (kg/day)	910	0.28	0.16
	Finisher	FinADG (kg/day)	707	0.89	0.13
Feed intake	Finisher	ADFI (kg/day)	704	2.22	0.33
		ADFD (min/day)	713	60.4	11.5
		FCR (kg/kg)	704	2.62	0.20
		RFI (kg)	703	0.04	0.12
Carcass	Carcass	CWT (kg)	653	91.3	10.0
		DRS (%)	651	77.6	2.0
		LYLD (%)	613	61.3	1.7
		CBF (mm)	615	16.9	3.8
		CLD (mm)	615	59.4	6.0

*Abbreviations: HS* subjective health score on a 1 to 5 healthy scale, *TRT* the number of treatments adjusted by the day that pigs stayed, *ADG* average daily gain, *ADFI* average daily feed intake, *ADFD* average daily duration, *FCR* feed conversion ratio, *RFI* residual feed intake, *CWT* carcass weight, *DRS* dressing proportion, *LYLD* lean yield, *CBF* carcass backfat, *CLD* carcass loin depth

### Population-level blood transcriptome data prior to challenge

Gene expression levels in blood collected from the 912 pigs in the qNUR, prior to their exposure to disease, were quantified by 3’mRNA sequencing with a globin block [11]. Descriptive statistics for the expression data are in Table 2. After trimming the raw reads, on average, 6.1 million (M) clean reads per sample were obtained. To assign reads to gene regions, annotation of 25,580 genes from the Ensembl SSC11.1.92 gene build was merged with gene annotation information obtained from Iso-seq data by Beiki et al. [20] (see Fig. S1). Use of the Iso-seq data resulted in 3’end extensions of genes that were annotated by Ensembl and the addition of another 12,491 genes. The merged annotation provided not only more accurate 3’end borders of genes, which increased the accuracy of gene expression quantification, but also data on additional genes for further downstream analysis. On average, 73.8% of all reads were uniquely mapped to the genome, of which 62.4% were assigned to gene coordinates, and 11.8% to the HBA and HBB gene regions. The latter were excluded from further analysis because globin block was applied in library construction. Only genes that had non-zero read counts in at least 80% of the samples (Fig. S2) were kept for further analyses, leaving data on 15,872 of the original 38,371 genes.

**Table 2:** Descriptive statistics for RNA quality and 3′ mRNA sequencing data on 912 pigs

Item	Mean	SD	Min	Max
RNA integrity number (RIN)	7.9	1.0	4.1	9.9
Total reads/sample (millions) ^a^	6.2	2.3	0.2	26.7
Aligned reads/sample (%) ^b^	98.6	1.4	67.2	99.5
Uniquely mapped reads/sample (%) ^b^	73.8	5.3	44.5	85.7
Gene reads (%) ^b^	62.4	10.5	30.5	85.7
HBA/HBB reads (%) ^b^	11.8	5.6	0.6	38.5
Non-globin reads (%) ^b^	50.6	7.5	27.0	70.7

*Abbreviations: SD* standard deviation, *Min* minimum, *Max* maximum, *HBA* hemoglobin subunit alpha, *HBB* hemoglobin subunit beta

^a^ The number of reads after trimming by Bbduk

^b^ The proportion to the total reads (%)

Normalized (by the trimmed mean of M values) and log2 transformed counts (Fig. S3) were adjusted for systematic effects using mixed linear models (Table S1) with (eWI) or without (eWO) accounting for the WBC composition of the sample. Comparing models with and without WBC composition, 17% (*n* = 2791) of genes showed a lower Bayesian Information Criterion (BIC) value for the model that included WBC composition, indicating that the observed level of expression of these genes was significantly affected by WBC composition. Almost all of these genes (*n* = 2715) were significantly associated with the proportion of lymphocytes, of which 876 were significant only for the proportion of lymphocytes. (Fig. S4). The numbers of genes whose expression was significantly (*q* < 0.10; Fig. S4) affected by only one of the other WBC types were 14, 4, 1, and 0 for monocytes, eosinophils, neutrophils, and basophils, respectively.

### Association of gene expression with phenotypes

Residuals of the expression values that were obtained for each gene for the 912 pigs from the eWO and eWI models, ResWO and ResWI, respectively, were used for quantitative analysis of associations of gene expression with concurrent (qNUR) and subsequent (cNUR and FIN) performance and resilience phenotypes. For most traits, there was no significant difference in their association with ResWO versus ResWI of gene expression based on a likelihood ratio test at *p* < 0.05 (Table S2). However, 193 genes were found to be significant for this test for feed conversion rate (FCR) and even more for MT (3816, 2033, and 3198 genes in the challenge nursery, the finisher, and across both phases, respectively). Note that the analyses for MT included only pigs that fell in the extremes in terms of mortality and treatment (died versus survived without treatment), which may affect the distributional assumptions of the likelihood ratio test.

To compare the sign and magnitude of associations with ResWO versus ResWI, and across phenotypes, estimates of the regression coefficients of phenotype on ResWO and ResWI were standardized by expressing them as the number of standard deviations of change in the phenotype that was associated with a one standard deviation change in expression. Signs of the estimates were reversed for resilience traits for which lower values are favorable (i.e. for TRT, MT, MOR, FCR, RFI, and back fat), such that a positive estimate always refers to a favorable change in the trait associated with an increase in expression. The resulting standardized estimates of regression coefficients obtained from regression on ResWO versus ResWI were highly correlated (0.92 to 1 across traits, Table 3), indicating that adjustment of expression for WBC did not result in large changes in associations of expression with phenotypes. Results for both ResWO and ResWI are presented (e.g. Fig. S5) but only results using ResWO will be described in the text.

**Table 3:** The number of genes with expression levels in blood of young healthy pigs that were significantly (*q* < 0.20) associated with observed phenotypes, with or without accounting for blood cell composition, the estimated number of genes that did not follow the null-hypothesis, and the relationship of adjusted estimates from two expression residuals across all genes

Trait measured during each phase ^a^	Number of genes from expression residuals with or without adjustment for cell composition			Correlation of estimates
	Without	With	With+Without ^b^	
***Quarantine Nursery***				
qNurHS1	395 (1656) ^c^	29 (1106)	395	0.92
qNurHS2	171 (982)	101 (0)	173	0.93
Growth rate	744 (3224)	830 (3357)	856	1.00
***Challenge Nursery***				
Health score	0 (0)	0 (0)	0	0.94
Treatment rate	0 (0)	0 (0)	0	0.96
Mortality + treatments	347 (3138)	170 (2956)	349	0.99
Mortality	7 (2592)	2 (2649)	7	1.00
Growth rate	0 (0)	0 (0)	0	0.98
***Challenge Finisher***				
Health score	0 (1048)	6 (1632)	6	1.00
Treatment rate	0 (176)	0 (48)	0	0.99
Mortality + treatments	0 (2014)	0 (1907)	0	0.99
Mortality	0 (2900)	0 (2787)	0	0.99
Growth rate	0 (0)	0 (0)	0	0.97
Feed intake	0 (270)	0 (0)	0	0.98
Feed intake duration	0 (0)	0 (0)	0	0.97
Feed conversion rate	422 (2768)	1 (1373)	422	0.95
Residual feed intake	0 (1892)	0 (1569)	0	0.97
***Overall Challenge***				
Treatment rate	0 (0)	0 (0)	0	0.98
Mortality + treatments	40 (2663)	0 (2152)	40	0.99
Mortality	1725 (5152)	1458 (5298)	1794	0.99
***Carcass***				
Carcass weight	0 (353)	0 (0)	0	0.96
Dressing proportion	0 (0)	0 (0)	0	0.96
Lean yield	42 (3912)	3 (2593)	42	0.97
Carcass backfat	18 (3555)	1 (2827)	18	0.97
Carcass loin depth.	0 (290)	0 (395)	0	0.99

Abbreviations: qNurHS1 and qNurHS2, health scores in the quarantine nursery

^a^ Health score was recorded by 1 (pigs in perfect health) and 0 (pigs with clinical signs); Mortality with treatments was recorded by 0 (pigs that survived without any treatment) and 1 (pigs that died during a given period); Mortality was recorded by 0 (survival) and 1 (death)

^b^ The number of genes that were significant for at least one of the models with or without accounting for blood cell composition

^c^ The number of significant genes (*q* < 0.20) from expression residuals with or without adjustment for cell composition and, within parentheses, the estimated number of genes that did not follow the null-hypothesis using the method descibed in [21]

Comparison of estimates of regression coefficients on gene expression between phenotypes recorded in each phase (Fig. S5) showed relationships that were consistent with the phenotypic correlations that were estimated between these traits in the whole NDCM population by Cheng et al. [19]. For example, in the challenge nursery, health score was phenotypically negatively correlated with both MOR (*r* = − 0.50) and TRT (*r* = − 0.30) [19], corresponding to the favorable relationship between standardized estimates of regression coefficients for these traits (Fig. S5 b).

Table 3 shows the numbers of genes that were significantly associated with phenotypes after correction for multiple testing (*q* < 0.20). The levels of gene expression were most strongly associated with traits that were measured during the qNUR, which is when the blood samples analyzed were collected. The number of significant genes for health scores (qNurHS1 and qNurHS2) and growth rate in the quarantine nurserywere 395, 173, and 856, respectively. A total of 14 genes were significant for all three traits recorded in the quarantine nursery (Table 4). Table 3 also provides the estimated number of genes that did not follow the null-hypothesis of no association with resilience, based on the method described in [21]. Sizable numbers of associated genes were estimated for most traits.

**Table 4:** Genes that showed significant association across all quarantine nursery traits in single-gene association analysis (*q* < 0.20)

Gene			Quarantine nursery traits			
Ensembl ID	Symbol	GO term of the direct biological process	Health score 1	Health score 2	Growth rate	Resilience
ENSSSCG00000003586	*EPB41*	blood circulation	WO+	BOTH+	BOTH+	
ENSSSCG00000008820	*TEC*	innate and adaptive immune response	WO-	WO-	BOTH-	
ENSSSCG00000009002	*TLR2*	inflammatory response, immune response	WO-	WO-	BOTH-	FCR^WO+^
ENSSSCG00000010025	*LIMK2*	spermatogenesis	WO-	WO-	BOTH-	
ENSSSCG00000010445	*ANKRD22*	–	WO-	WO-	BOTH-	
ENSSSCG00000012880	*CPT1A*	regulation of insulin secretion, eating behavior	BOTH-	WO-	BOTH-	
ENSSSCG00000013065	*ASRGL1*	proteolysis	WO-	WO-	BOTH-	FCR^WO+^
ENSSSCG00000013556	*ADGRE1*	adaptive immune response	WO-	WO-	BOTH-	
ENSSSCG00000017126	*NARF*	–	WO+	BOTH+	BOTH+	
ENSSSCG00000020872	*–*	–	BOTH+	BOTH+	BOTH+	LYLD ^WO+^
ENSSSCG00000033146	*CD163*	acute-phase response, viral entry into host cell	WO-	WO-	BOTH-	NurMT^BOTH-^AllMOR^WO-^
ENSSSCG00000033190	*–*	–	WO+	BOTH+	BOTH+	
ENSSSCG00000033703	*FAM111A*	defense response to virus	WO-	WO-	BOTH-	NurMT^BOTH-^FCR^WO+^
ENSSSCG00000035182	*SDR42E1*	steroid biosynthetic process	WO-	WO-	BOTH-	

Abbreviation: WO and WI, the significant association (q < 0.20) between observed phenotypes and gene expression levels with or without accounting for with blood cell composition, respectively; BOTH, the significant associations of both expressions with and without accounting for blood cell composition, with observed phenotypes; + and -, the favorable and unfavorable direction of relationship with observed phenotypes, respectively; FCR, feed conversion rate; LYLD, lean yield; NurMT, mortality with treatment during challenge nursery; AllMOR, mortality across whole period

For traits that were recorded during the challenge phase, most significant associations were found for MOR, MT, and for FCR (Table 3). Gene expression was more strongly associated with mortality across the challenge nursery and finisher (AllMOR) than with mortality within each phase (NurMOR or FinMOR): 1794 genes were significantly associated with AllMOR, of which only 7 genes were associated with NurMOR and none with FinMOR. In contrast, the number of genes that were significantly associated with MT was higher in the challenge nursery(*n* = 349) than across the challenge nurseryand finisher (*n* = 40). Among feed-related traits, only FCR showed a significant association with gene expression levels (*n* = 422). The numbers of genes whose expression was significantly associated with carcass traits were 42 for lean yield and 18 for back fat thickness.

Among the 14 genes that showed significant associations with all three qNUR traits (Table 4), five genes were also significantly associated with subsequent resilience traits (*q* < 0.20): *CD163*, which encodes the receptor for PRRS virus entry and replication in alveolar marcophages [22], was associated with NurMT and AllMOR (estimates of associations with all phenotypes are in Table S3); *family with sequence similarity 111 member A* (*FAM111A*) was associated with MT in the challenge nursery and with FCR (only measured in the finisher); *toll like receptor 2* (*TLR2*) and *asparaginase and isoaspartyl peptidase 1* (*ASRGL1*) were associated with FCR; and *PDZK1 interacting protein 1* (ENSSSCG00000020872) was associated with lean yield. The signs of the associations of these genes with traits reflected the phenotypic correlations between the traits [19], with higher expression of *CD163* and *FAM111A* being unfavorably associated with both qNUR traits and with MT in the cNUR; higher expression of *FAM111A*, *TLR2*, and *ASRGL1* was unfavorably associated with qNUR traits and favorably with FCR; and higher expression of ENSSSCG00000020872 was favorably associated with qNUR traits and with lean yield.

### Gene set enrichment analysis of expression associations with phenotypes

**Fig. 2:**
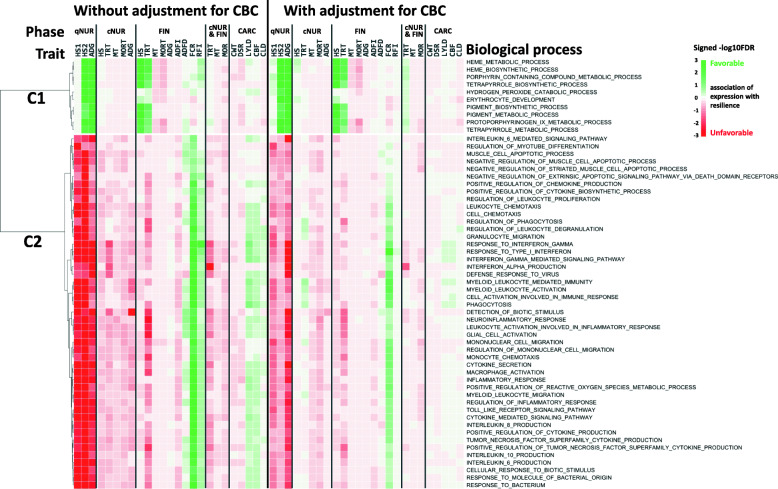
Biological processes (*n* = 57) that were significantly (FDR < 0.001) enriched among genes ranked based on the magnitude of the association of their expression with at least one quarantine nursery trait based on gene set enrichment analysis and the signed significance (−log10(FDR)) of the enrichment of these biological processes with disease resilience traits, with or without adjustment of gene expression for cell composition. Green/Red = an increase in expression of genes in that biological process is favorably/unfavorably associated with disease resilience (e.g. less/more mortality, treatments, higher/lower growth rate, feed intake, etc.)

**Fig. 3:**
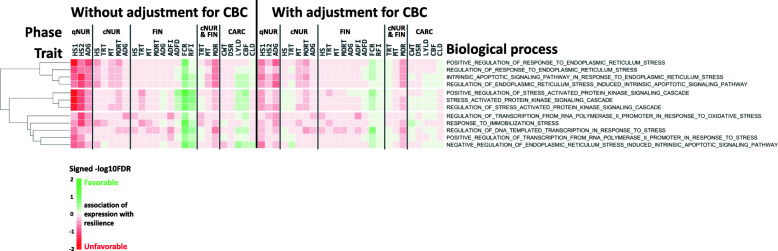
Stress-related biological processes (*n* = 12) that were significantly (FDR < 0.20) enriched among genes ranked based on the magnitude of the association of their expression with at least one quarantine nursery trait based on gene set enrichment analysis and the signed significance (−log10(FDR)) of the enrichment of these biological processes with disease resilience traits, with or without adjustment of gene expression for cell composition. Green/Red = an increase in expression of genes in that biological process is favorably/unfavorably associated with disease resilience (e.g. less/more mortality, treatments, higher/lower growth rate, feed intake, etc.)

**Fig. 4:**
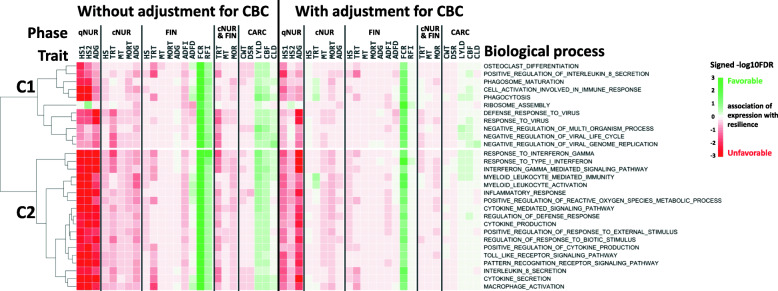
Biological processes (*n* = 29) that were significantly (FDR < 0.001) enriched among genes ranked based on the magnitude of the association of their expression with at least one feed efficient trait based on gene set enrichment analysis and the signed significance (−log10(FDR)) of the enrichment of these biological processes with disease resilience traits, with or without adjustment of gene expression for cell composition. Green/Red = an increase in expression of genes in that biological process is favorably/unfavorably associated with disease resilience (e.g. less/more mortality, treatments, higher/lower growth rate, feed intake, etc.)

**Fig. 5:**
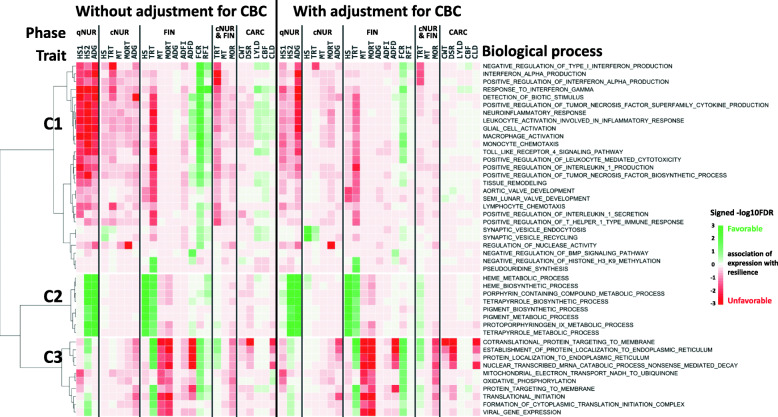
Biological processes (*n* = 45) that were significantly (FDR < 0.025) enriched among genes ranked based on the magnitude of the association of their expression with at least one resilience trait except for feed efficiency traits based on gene set enrichment analysis and the signed significance (−log10(FDR)) of the enrichment of these biological processes with disease resilience traits, with or without adjustment of gene expression for cell composition. Green/Red = an increase in expression of genes in that biological process is favorably/unfavorably associated with disease resilience (e.g. less/more mortality, treatments, higher/lower growth rate, feed intake, etc.)

The limited statistical power to detect associations of the expression of an individual gene with a phenotpe can be overcome by analyzing associations for groups of genes. Here, associations across genes were leveraged by GO-term gene set enrichment analysis (GSEA) of association results obtained for all 15,872 genes. For this purpose, for each trait, genes were ranked based on their standardized regression coefficient estimates and analyzed for GO-term enrichment using the GSEA_4.0.3 software [23]. Significance (−log10(FDR)) and direction of associations of the expression of genes with a given GO term for each trait are shown in heat maps in Figs. 2, 3, 4 and 5. GO terms in the heat maps were ordered by hierarchical clustering based on the signed significance (−log10(FDR)) of their enrichment across traits and across associations with or without adjustment of gene expression residuals for WBC composition. The enriched terms for gene expression residuals with or without adjustment for WBC composition showed a similar trend in the direction of associations across traits.

Figure 2 shows two clear clusters of biological processes (*n* = 57) that were significantly (FDR < 0.001) enriched for associations with at least one qNUR trait. Biological processes in the first cluster, which included heme metabolism-related terms and the hydrogen peroxide catabolic process, were favorably associated with qNurHS2 and with growth rate in the qNUR, as well as with HS and TRT in the finisher. The second cluster showed unfavorable associations with qNUR traits and also with phenotypes under challenge, except with FCR and RFI. This indicates that increased expression of genes in these biological processes in blood in the quarantine nursery was associated with poorer performance, both before and during the challenge. Predominant in this cluster were immune-related terms such as response to virus or bacterium, myeloid leukocyte activation, phagocytosis, inflammatory response, cytokine production (interleukin, interferon-alpha/−gamma, toll like receptor, tumor necrosis factor), and cell chemotaxis. This cluster also included biological processes related to muscle apoptosis.

Although pigs were expected to be free of major diseases at the time of blood collection, they had been exposed to non-disease stressors, such as weaning, transportation, and mixing. Thus, genes involved in stress-related biological processes were further examined in terms of their association with traits recorded in the quarantine nursery. In total, 12 stress-related biological processes tended to be associated with qNUR traits (FDR < 0.20) (Fig. 3). These processes showed a similar pattern of associations with phenotypes under challenge as cluster 2 in Fig. 2. Similar to the immune-related terms in cluster 2 of Fig. 2, the significant stress-related terms showed an opposite direction of associations for FCR and RFI.

Figure 4 shows biological processes that were significantly enriched for at least one feed efficiency trait at FDR < 0.001. Most biological processes that were significantly enriched among genes with favorable associations with feed efficiency traits were related to immune response and showed unfavorable associations with other resilience traits. However, the term of ribosome assembly in cluster 1 was favorably enriched not only for feed efficiency traits, with limited impacts on other resilience traits, but also for HS2 during the qNUR.

Figure 5 shows biological processes that were significantly enriched for at least one phenotype recorded during the challenge, other than FCR and RFI (FDR < 0.025). Among these, three clusters were identified with distinct patterns of significance across resilience traits. The signs of the associatons for enriched terms within each cluster were similar for ResWO and ResWI, although their significance levels differed, depending on the cluster. Cluster 1 included immune-related GO terms that were unfavorably associated with traits in both the quarantine nursery (growth rate and HSs) and under challenge, except for FCR and RFI. Cluster 1 also included GO terms for aortic/semi-lunar valve development and regulation of nuclease activity, with unfavorable associations with resilience traits. Cluster 1 also included GO terms with favorable associations with resilience traits, such as HS and TRT in the challenge nursery(synapse vesicle endocytosis, BMP signaling pathway), and TRT in the finisher (histone methylation, and pseudouridine synthesis). GO terms in cluster 2 contained heme metabolism-related terms that were shown in Fig. 2 to be favorably associated with HS and TRT in the finisher, both with and without adjustment of gene expression for WBC composition, with weak and non-significant unfavorable associations with other resilience traits. Cluster 3 included GO terms that were unfavorably associated with most resilience traits, as well as with carcass traits, but that were favorably associated with FCR and TRT in the finisher. This cluster included protein localization, nonsense-mediated decay, and viral gene expression. In contrast to most GO terms in cluster 1, GO terms in cluster 3 had weak associations with qNUR traits.

## Discussion

### Blood transcriptome of young healthy pigs

The objective of this study was to investigate the biological basis of gene expression patterns of young healthy pigs that are associated with their future disease resilience, rather than to understand host response to specific pathogens. Most previous studies using blood transcriptome profiling in relation to disease used artificial infection of animals with specific pathogens and blood samples collected at multiple time points to quantify gene expression and/or pathogen burden in the host [5–10]. Compared to those studies, the current study is novel in several respects. First, we applied a natural polymicrobial disease challenge model to cover common pathogens seen in commercial pig farms and natural exposure to these pathogens, instead of applying an artificial infection challenge with one or a limited number of pathogens. The objective was to mimic a commercial environment with high disease pressure. Detailed phenotypes related to disease resilience were collected on a large number of animals, including mortality, health treatments, health scores, growth rate, feed efficiency, and carcass traits. Second, blood for gene expression analysis was collected prior to exposure to the natural disease challenge in order to identify gene expression patterns in young healthy pigs that were associated with subsequent resilience. As a result, the gene expression experimental design did not include a treatment group and the focus of the analyses was to evaluate differences in gene expression between healthy animals and how these differences were related to subsequent disease resilience following exposure to the polymicrobial challenge. Last, we applied a quantitative association approach for the gene expression analysis based on the population-level blood transcriptome data from 912 biological replicates, as this was not a treatment versus control design.

### Adjustment of gene expression patterns for cell composition

Cellular heterogeneity is an issue for bulk RNA-seq analysis of blood samples and several deconvolution methods have been proposed to predict and account for cell composition [15, 16]. Dong et al. [24] used predictions of cell composition based on the RNA-seq data to account for cellular heterogeneity in differential gene expression analysis in tonsil for persistent porcine reproductive and respiratory syndrome virus (PRRSV) infection. Cellular heterogeneity of the tonsil samples was found to have a large effect on gene expression levels. In the current study, we used WBC composition data to account for cell type proportion heterogeneity across blood samples; residuals from a mixed linear model with or without accounting for the proportions of six cell types were used for analysis of associations with recorded phenotypes. In total, the expression levels of 2791 genes were significantly affected by WBC composition (lower BIC and *q* < 0.10; Fig. S4). Because of this, associations of gene expression with disease resilience were evaluated both with and without adjustment of gene expression for WBC composition (ResWI and ResWO). In general, however, the estimates of regression coefficients on ResWO versus ResWI were highly correlated (Table 3) and association results with disease resilience traits were minimally impacted by adjustment for WBC composition (Table S2). However, GSEA analysis of association results for ResWO showed higher significance levels for enrichment of immune-related biological processes than results for ResWI (Fig. 5). This implies that WBC proportions do not only affect gene expression levels in blood but can also be directly associated with the measured phenotypes. Bai et al. [25], who analyzed associations of WBC proportions with disease resilience using data from 42 batches of the NDCM, reported that WBC composition of blood collected during the quarantine nursery(at the same time as used here) did not differ significantly between animals that differed in resilience. To add to these results, and to confirm the effect of WBC composition on qNUR traits, we investigated the relationship of these WBC proportions with qNUR traits, using data from the whole NDCM population (*n* = 2819). Results revealed that the proportion of lymphocytes was significantly associated with health scores in the quarantine nursery(*p* < 0.01; data not shown), supporting the effects of WBC composition on gene expression levels quantified during the same phase. Adjustment of gene expression levels for WBC composition (ResWI) resulted in the identification of additional significant genes (*q* < 0.20) in the single-gene association analyses (Table 3) and several non-immune related biological processes showed stronger associations with resilience traits in the GSEA results (Fig. 5), including cotranslational protein targeting the membrane and protein localization to the endoplasmic reticulum. These findings support the usefulness of evaluating the effects of adjustment of gene expression for cell composition when conducting transcriptome profiling in heterogeneous tissues.

### Associations of gene expression with phenotypes collected on young healthy pigs

Pigs were sourced from high health multiplier farms and were kept in a biosecure environment prior to the disease challenge. During the 3-week acclimation period in the qNUR, none of the pigs used in this study (*n* = 912) received a therapeutic treatment. In addition, most pigs were in good health 2 weeks into the quarantine nursery(86% of pigs had a HS of 5), although some lower health scores were observed 6 days after arrival in the quarantine nursery(16% with a score of 4), which is when blood samples for RNA-seq were taken. The expression of 1207 genes was associated (*q* < 0.20) with health scores and/or growth rate in the qNUR, as shown in Table 3. The expression of five immune-related genes was unfavorably associated with all three qNUR traits (Table 4): *tec protein tyrosine kinase* (*TEC*), *TLR2*, *adhesion G protein-coupled receptor E1* (*ADGRE1*), *CD163*, and *FAM111A* (Table 4). TLR2 recognizes many bacterial, fungal, viral, and certain endogenous substances, and is involved in activation of innate immunity [26, 27]; *ADGRE1* is the marker gene for myeloid differentiation in pigs [28]; expression of *CD163* is increased during infection with *A. pleuropneumoniae* [29] and *H. parasuis* [30]; *FAM111A* plays a role in inhibiting viral genome replication [31]. In addition, GSEA showed that genes for which an increase in expression was unfavorably associated with qNUR traits were enriched for GO terms related to immunity (FDR < 0.001; Fig. 2) and stress response (FDR < 0.20; Fig. 3), indicating pigs that had higher expression levels of genes involved with immune- and stress-related biological processes had lower health scores and growth rates in the qNUR. This suggests that the differences in blood transcriptome that were observed in the quarantine nurserymay reflect responses to subclinical or minor infectious disease and/or to non-infectious stressors such as weaning, transportation, and mixing. For example, He et al. [32] reported that the unfolded protein response pathway related to endoplasmic reticulum stress was activated in the small intestine of pigs due to weaning. As reviewed by Gimsa et al. [33], the immune system and stress response are closely associated in pigs.

The *CD163* gene, one of the genes that was significantly associated with all three qNUR traits, encodes the receptor required for PRRS virus to attach to and infect macrophages [22]. Pigs that had higher *CD163* expression in blood on average had poorer health scores and lower growth rates in the qNUR. The *CD163* gene encodes a scavenger receptor that leads to the removal of the haptoglobin-hemoglobin complex from blood [34]. The *CD163* gene is expressed predominantly in monocytes and macrophages in pigs [35] and soluble CD163 suppresses proliferation of lymphocytes [36]. Cell type-specific expression patterns of *CD163* are consistent with the significant effects of WBC composition on *CD163* expression observed in our study (*q* < 0.001; Table S4), which showed positive and negative associations of *CD163* expression levels with the proportions of monocytes and lymphocytes, respectively. In addition, *CD163* expression residuals showed significant associations with growth rate in the quarantine nurseryand with MT in the challenge nursery(*q* < 0.20, Table 4) both with and without accounting for WBC composition, indicating that the higher expression of *CD163* may come from a combination of a higher proportion of monocytes, as well as from activation of *CD163* gene expression within monocyte cells. The level of soluble CD163 has been suggested as an indicator for autoimmune disorders such as systemic lupus erythematosus [37]. CD163 also functions as a sensor of innate immune response and inflammation by binding some pathogenic bacteria [38] and viruses, such as swine fever virus [39] and PRRS virus [22]. *CD163* knockout pigs created by gene editing are completely resistant to the PRRS virus [40–42]. In addition, Dong [43] showed that natural variants in the *CD163* gene are associated with resistance to PRRS. Based on the biological features of *CD163*, healthy pigs with abnormally high *CD163* levels due to over-expression or high levels of monocytes may exhibit autoimmune abnormalities, abnormal iron metabolism, or subclinical infections, which could trigger excessive macrophage activation. This can lead to lower resilience when these pigs are exposed to disease, because many viral and bacterial pathogens, including PRRSV, PCV2, *Salmonella spp*, and *Mycoplasma hypopneumoniae*, replicate within macrophages [35]. This is supported by the unfavorable associations of *CD163* expression residuals (without or with WBC composition adjustment) prior to exposure to disease with multiple resilience phenotypes in the challenge nursery(*p* < 0.06) (Table S3).

### Associations of gene expression prior to exposure with phenotypes collected after exposure

Only some phenotypes collected during the challenge (MOR, MT, and FCR) showed significant associations with gene expression in blood collected prior to the challenge for a substantial number of genes (Table 3), likely because of limited statistical power of the single gene association analyses. Compared to MOR, MT showed a greater number of significant genes (q < 0.20, Table 3), although MT results were based on less data. Compared to contrasting pigs that survived versus died, MT compared pigs that survived without treatment to those that died, providing a clearer contrast. The level of expression in blood prior to exposure to disease of the *guanylate binding protein 5* (*GBP5*) gene, a candidate gene for host response to PRRS virus infection [44–46], was not significantly associated with resilience traits but tended to be favorably associated with mortality in the challenge nursery(both for ResWO and ResWI, *p* = 0.02). Despite the small number of significant genes, the number of genes that were estimated to not follow the null-hypothesis of no association with resilience was sizeable for most traits (Table 3), including for traits that did not show any significant associations. This suggests that blood gene expression profiles in healthy pigs prior to the challenge were associated with their future disease resilience, although few genes showed significant associations in the single gene analyses, because of limited power. This was confirmed by the GSEA results, which showed that many GO terms were enrichment among genes whose expression was associated with resilience traits. The GSEA analysis essentially evaluates the relationship of groups of genes, based on GO terms, with recorded phenotypes, rather than one gene at a time. In the following, we will first discuss GO terms that were enriched among genes whose expression was associated with both phenotypes collected prior to and during the challenge, followed by discussion of GO terms that were associated only with phenotypes collected during the challenge.

#### Biological processes associated with phenotypes collected before and during the challenge

Biological processes related to the immune/stress responses and heme metabolism (clusters 1 and 2, respectively, in Fig. 5) were associated not only with phenotypes in the quarantine nursery but also with responses under disease challenge, with biological processes related to immune/stress response in cluster 1 showing unfavorable associations with phenotypes, and biological processes related to heme metabolism in cluster 2 showing favorable associations. It is generally accepted that an increase in expression of immune-related genes following exposure to pathogens is associated with higher disease resistance. This hypothesis has been supported by previous gene expression studies with artificial infection that targeted differences in the resistance or susceptibility between breeds in pigs [47] or between inbred lines of chickens [48, 49]. However, our results suggest that piglets that had greater expression of immune- and stress-related genes in blood prior to exposure tended to be less resilient to disease upon exposure. It should be noted, however, that the current study focused on differences in gene expression prior to disease exposure, while most previous studies have analyzed changes in gene expression after or during infection as a response to disease. However, the expression of genes with GO terms that were enriched for immune- and stress-related biological processes also had an unfavorable association with phenotypes measured in the quarantine nursery (growth rate and health scores). This suggests that the expression levels of these genes reflect responses to several stressors that the pigs were exposed in the week prior to blood collection, including weaning, transportation, and new feed ingredients. It is well known that weaning and transportation cause acute stress in pigs [33, 50]. In addition, exposure to new feed ingredients may tax mucosal immune response [51]. This suggests that pigs that are more impacted by those stressors and, as a result, have greater expression of those genes, are also more susceptible to disease under a severe challenge.

It is also notable that the GO terms in cluster 1 of Fig. 5 tended to be favorably associated with FCR (measured only in the finisher) and, to a lesser extent, with carcass traits. This result is, however, consistent with the observed phenotypic correlations of FCR with growth rates in the different phases across 50 batches of the NDCM, which was negative (− 0.28) for ADG in the finisher, which was as expected, but positive with ADG in the quarantine nursery (0.22) and in the challenge nursery (0.35). However, the biological process of ribosome assembly had a favorable association with feed efficiency traits, without deleterious impacts on other resilience traits (Fig. 4). This implies that the ribosome assembly process could be a target for improving feed efficiency under challenge without decreasing resilience. This is consistent with results of Bottje et al. [52], who reported that proteins that showed greater levels in breast muscles of chickens with high feed efficiency were enriched for the ribosome assembly process.

In contrast to immune-related terms in cluster 1 of Fig. 5, pigs with higher expression of genes that belonged to the heme metabolism-related biological processes in cluster 2 were associated with better health status and growth rate prior to exposure disease and also with better health status and lower treatment rate in the finisher. Note that disease pressure was lower in the finisher than in the challenge nursery and many pigs recovered from disease in the finisher, suggesting that the impact of heme metabolism-related biological processes may be limited during severe disease pressure or may reflect the ability to recover from the diseases. Heme, a ferrous iron protoporphyin IX complex, is involved in many essential biological processes as a prosthetic group in diverse hemoproteins (reviewed by Lin and Wang [53]). Previous studies in pigs revealed that heme iron supplement is favorably associated with body weight and mortality [54] and counteracts iron deficiency anemia [55]. Jointly, these results suggest that the expression of genes related to heme metabolism, especially synthesis, may have favorable impacts on disease resilience.

#### Biological processes only associated with phenotypes collected during the challenge

Biological processes in cluster 3 in Fig. 5 showed stronger associations with phenotypes collected during the challenge than prior to the challenge. These included processes related to protein localization and viral gene expression, which were unfavorably associated with phenotypes after exposure. Viruses typically enter host cells via attachment factors and/or viral receptors, after which the virus particles are localized to an appropriate site in the cell for viral gene expression and genome replication, which is a part of the general life cycle of viruses (reviewed by Ryu [56]). Transcription of most DNA and RNA viruses takes place in the nucleus and in the cytoplasm of the host cell, respectively (reviewed by Gale et al. [57]). Park et al. [58] reported that subcellular localizations of viral proteins were directly correlated with disease phenotypes in humans. Furthermore, viruses rely on host transcription and translation machinery to propagate and to produce progeny viruses (reviewed by Gale et al. [57]). This suggests that gene sets related to protein localization and viral gene expression have biological implications for response to disease. As noted, GO terms in cluster 3 showed limited associations with phenotypes prior to the challenge compared with the terms in clusters 1 and 2, suggesting that the expression levels observed for the genes in cluster 3 were baseline. Based on this, we can hypothesize that young healthy pigs that have a lower baseline expression of genes in cluster 3 are more resilient when exposed to disease.

## Conclusions

This study on the integration of quantitative analysis of population-level blood transcriptome data prior to exposure to disease with performance prior to and after exposure provides insight into the biological basis behind gene expression patterns in blood of young healthy pigs and how this is associated with their concurrent performance and with their resilience when exposed to disease. Our results suggest that gene expression in blood of recently weaned piglets in high-health herds in part reflects their susceptibility and response to various stressors that they are exposed to, even in biosecure conditions, such as weaning, transport, and mixing, as well as the effects of exposure to new dietary ingredients, which may affect mucosal immune response, and that these gene expression patterns are phenotypically associated with disease resilience. This included genes related to immune and stress responses, and heme metabolism, which are, therefore, candidate genes for stress and disease resilience. Our results also identified biological processes based on gene expression in blood of young healthy pigs that were associated with disease resilience but not with performance prior to exposure. These included host machinery genes involved in viral translocation, transcription, and translation. This implies that variation in the baseline expression of these genes prior to exposure could have an impact on disease resilience. Single gene association analyses revealed that higher expression of the *CD163* gene in blood prior to the disease challenge was closely associated with mortality after exposure, which included the PRRS virus. Taken together, significant relationships between blood transcriptome in healthy weaned piglets and their resilience following exposure to a natural polymicrobial disease were identified, implicating their possible use as early disease resilience indicators, at least at the phenotypic level. Subsequent studies will focus on genetic analyses of these data.

## Methods

### Ethical statement

The protocol of this project was approved by the Animal Protection Committee of the Centre de recherche en sciences animales de Deschambault (15PO283) and the Animal Care and Use Committee of the University of Alberta (AUP00002227), and was based on the Canadian Council on Animal Care guidelines (CCAC; https://www.ccac.ca/en/certification/about-certification). Comprehensive supervision of animal care was provided by the Centre de développement du porc du Québec (CDPQ) and the herd and project veterinarians. If needed, pigs in the natural disease challenge were humanely euthanized (*n* = 87). Following CCAC guidelines, electrocution was used in the nursery and cranial captive bolt during the finisher period. Pigs that reach slaughter weights were stunned by electrocution at a commercial slaughter facility to enter the food chain, followed by exsanguination, using standard approved industry protocols.

### Study design

A total of 912 pigs in 15 batches from the NDCM were used in the current study of population-scale transcriptome analysis targeting disease resilience (Fig. 1). Details of the NDCM were described in [18, 19]. Briefly, single-sourced batches of 60 or 75 healthy weaned barrows (Yorkshire x Landrace) from one of seven breeding programs, which provided batches in rotation, were transported to an experimental facility at ~ 21 days of age. They were acclimated for 3 weeks in a healthy quarantine nursery (phase 1) and then moved to a nearby natural disease challenged nursery (phase 2; 3 ~ 4 weeks) and finisher (phase 3; up to slaughter at ~ 180 days of age). The challenge nursery-finisher aimed to mimic a commercial farm with high disease pressure. Pigs were exposed to a natural polymicrobial disease challenge that included common viruses and bacteria that are present across commercial farms, including PRRSV, porcine circovirus type 2, *M. hyopneumoniae*, *Streptococcus suis*, and others. Pigs received no vaccinations, except for a procine circovirus type 2 vaccine (Circoflex, Boehringer Ingelheim, St. Joseph MO), which was given at the same time that blood for RNA-seq and WBC composition were collected.

### Measurements of resilience

Performance traits relevant to disease resilience, including subjective health scores, therapeutic treatment rates, mortality, growth rate, feed efficiency, and carcass traits were collected. Subjective health scores (HS) were assigned to each pig at four time points; on the day of blood collection at day 5 post entry into the quarantine nursery (qNurHS1), at the end of the quarantine nursery (qNurHS2), two weeks post entry into the challenge nursery (NurHS), and 6 weeks post entry into the challenge nurseryat the finisher (FinHS). Health scores were recorded on a 1 to 5 scale, as described in [19] (1 = severe clinical signs with wasting; 2 = severe clinical signs without wasting; 3 = mild to moderate clinical signs with or without wasting; 4 = mild clinical signs without wasting or light wasting without any other clinical signs; 5 = in perfect health). Health scores were converted into binary variables (0/1; 1 = pigs in perfect health; 0 = others) for the single gene association analysis. The number of individual therapeutic treatments was adjusted to 27 days for the challenge nursery (cNurTRT), to 100 days for the challenge finisher (cFinTRT), and to 180 days for whole challenge period (AllTRT), as described in [19]. Pigs that exhibited clinical signs of pneumonia, diarrhea, lameness, arthritis, meningitis, dermatitis, pallor, lethargy, weight loss, unthriftiness, cyanosis, or conjunctivitis were treated with 1 of 10 antibiotics based on a treatment protocol that outlined primary and secondary (if needed) treatments for each ailment. For some clinical signs, one of two anti-inflammatory drugs was also administered, while batch-level water medication was used during periods of severe illness, including a water-soluble anti-inflammatory drug to treat batches that suffer from severe respiratory disease (primarily PRRS). All individual and group treatments were recorded [18]. Mortality (recorded as 0 for pigs that survived and 1 for pigs that that died) was recorded during the challenge nursery (cNurMOR), the finisher (cFinMOR), and across the challenge nurseryand finisher (AllMOR). Mortality combined with individual therapeutic treatment (MT, recorded as 0 for pigs that survived with no individual therapeutic treatment, 1 for pigs that that died, and missing for all other pigs) was defined for the challenge nursery (cNurMT), the finisher (cFinMT), and across the challenge nurseryand finisher (AllMT). Average daily gain (ADG) was computed as described in [19] for the quarantine nursery (qNurADG), the challenge nursery (cNurADG), and for the finisher (cFinADG). Average daily feed intake (ADFI), average daily feeding duration (ADFD), FCR, and RFI were recorded in the finisher, as described in [19]. Carcass weight (CWT), dressing proportion (DRS), lean yield (LYLD); carcass backfat (CBF), and carcass loin depth (CLD) were recorded as described in [19].

### Blood RNA extraction and white blood cell count measurement

Blood samples were collected in the quarantine nurseryat ~ 27 days of age, using Tempus Blood RNA Tubes (Thermo Fisher Scientific, USA) and then stored at − 80 °C until RNA extraction. The RNAs were isolated using Preserved Blood RNA Purification Kit I (Norgen, Canada) according to the manufacturer’s instructions. The RNA integrity number (RIN) of each extracted RNA was assessed by the 2100 Bioanalyzer (Agilent Technologies, USA) using the Eukaryote total RNA 6000 Nano kit. The RIN score was on average 7.9 and ranged from 4.1 to 9.9 (Table 2). WBC differentials were quantified on whole blood samples in K2 ethylenediaminetetraacetic acid (EDTA) tubes (Thermo Fisher Scientific, USA) taken at the same time, using the flow cytometry-based hematology analyzer (ADVIA®2120i Hematology System, Simens Healthineers, Germany) according to the manufacturer’s instructions [59]. The log2 transformed proportion of each WBC type was used to adjust gene expressions levels for blood cell composition (see later).

### 3′ mRNA sequencing with globin blocking

RNA-seq libraries were generated from ~ 500 ng of total RNA, using the QuantSeq 3′ mRNA-Seq Library Prep Kit FWD for Illumina with the RNA Removal Solution Globin Block, *Sus scrofa*, according to the manufacturer’s protocol (Lexogen, Austria), as described by Lim et al. [11]. The first-strand cDNA was synthesized by reverse transcription with oligo-dT priming. Prior to second strand synthesis, porcine HB-specific oligonucleotide mixtures that are present in the globin block bind to the first strands that were generated from mRNAs of HBA and HBB, thereby preventing second strand synthesis. The constructed QuantSeq libraries were multiplexed using mRNA from up to 96 samples and sequenced with single-end 50 bp using the Illumina HiSeq 3000 Sequencing System (Illumina, USA). Each library was sequenced on two lanes and the sequence reads obtained from the two lanes were combined.

### RNA-seq read processing

The raw QuantSeq reads were first processed using BBDuk (https://jgi.doe.gov/data-and-tools/bbtools/bb-tools-user-guide/bbduk-guide/) to trim the adapter sequences, poly-A tails, and low-quality bases, and to filter out reads with a length less than 20 bp after trimming. Read quality before and after trimming was assessed using FASTQC 0.11.5 [60]. Then, trimmed reads were mapped to the *Sus Scrofa* reference genome sequence (SSC11.1; Ensembl, http://www.ensembl.org/) using STAR 2.5.3a [61]. To overcome the high sensitivity of 3’mRNA sequencing to 3’end gene annotation, we added 3’UTR information and used windows for 3’end extension and for the exon region. For gene annotation, we used both the pig reference genome sequence assembly (Ensembl release 92; ENS) and the Iso-seq based annotation (ISO) developed by Beiki et al. [20], which includes information on 3’end extensions. In addition, to obtain read counts for *GBP5*, which is not annotated in Ensembl SSC11.1, three (ENSSSCT00000065307, ENSSSCT00000060466, and ENSSSCT00000044130) of nine transcripts that were annotated as *guanylate binding protein 1* (*GBP1*) were assigned to *GBP5* based on the location of the WUR SNP (rs80800372) and of the putative causative *GBP5* intronic SNP (rs340943904), as identified by Koltes et al. [46]. The final gene annotation file contained 38,371 genes, comprising 14,815 ENS-specific genes, 12,491 ISO-specific genes, and 11,065 genes that were present in both the Ensembl and the ISO-seq annotated lists of genes (Fig. S1). Then, two modified gene annotation files were generated, with 3’end extension windows up to 1 kb for each transcript and with all exons included, or only the last exon (Fig. S6). Based on these two annotation files, a QuantSeq-specific read count method was implemented to count reads for each gene, using an in-house Python script (Fig. S7) using HTseq modules [62]. First, unique-mapping reads were counted for each gene based on the annotation file that included all exons and the 3’end extension window. Then ambiguous unique-mapping reads and multiple-mapping reads were counted if they were mapped to the annotation file that included only the last exon and the 3’end extension window.

### Gene expression normalization and standardization

Reads that mapped to the globin genes HBA and HBB and to genes that had a zero-count in more than 80% of the samples were filtered out. Remaining read counts were normalized across the 912 samples by the trimmed mean of M values (TMM) using the EdgeR package in R [63]. Then, a log2 transformation was applied to the normalized counts plus 1 to obtain scaled expression values for further analyses.

The normalized and scaled expression values were adjusted for nuisance factors using mixed linear models with (eWI) or without (eWO) accounting for WBC composition (Table S1), separately for each gene. The eWO model included batch as a fixed effect, pen in the quarantine nursery as a random effect, and RIN and age (days) when pigs entered the quarantine nursery as covariates. The eWI model additionally included the log2 transformed proportions of six WBC types as covariates: lymphocytes, monocytes, neutrophils, eosinophils, basophils, and large unstained cells. Bayesian information criterion values between the eWO and eWI models were compared to evaluate the significance of WBC composition on the observed expression of each gene. The resulting standardized residuals of the single gene expression values from the eWO or eWI models were use in subsequent analyses of associations with phenotypes.

### Association analysis of gene expression with phenotypes

To identify associations of gene expression of young healthy piglets in blood with recorded phenotypes, both in the quarantine nurseryand during the challenge, forward and reverse analyses were applied for the analysis of continuous and categorical resilience traits, respectively. In the forward analysis, for growth rate, TRT, feed intake, and carcass traits, residuals of gene expression from the eWO and eWI models were fitted as covariates, one-by-one, in a mixed linear model for analysis of each trait. The models used in the forward analysis are summarized in Table S1. Briefly, all models included batch as a fixed effect, age of entry into the quarantine nurseryand expression residuals of a single gene as covariates, and litter and pen as random effects. In the model for carcass traits, slaughter date was added as a fixed effect, and age and weight at slaughter were added as covariates. For phenotypes across the challenge nurseryand finisher, such as AllTRT, only pen in the challenge nurserywas fitted since the pigs that died in the nursery did not have a finisher pen. For binary traits, i.e. health scores, mortality, and mortality combined with treatment, logistic regression analyses were attempted but failed to converge in a number of cases. For these traits, instead, a reverse analysis was applied, in which the expression residuals of a gene were used as the response variable and the binary trait was included as a fixed effect in the association model. The resulting estimates of the binary trait effect were then converted to estimates of the regression of the resilience on expression using the variances of the binary trait and expression residuals.

All mixed linear models were fitted in R using the lmer function from the lme4 package [64]. To compare the goodness-of-fit between the association models with gene expression residuals from the eWO and eWI models, a likelihood ratio test with 1 degree of freedom was conducted for each gene. For the reverse analysis, this test was conducted by fixing the estimate of the effect of the binary trait when analyzing residuals with (without) WBC composition adjustment to the estimate obtained from analysis of residuals without (with) WBC composition adjustment by subtracting that estimate from the response variable. For multiple testing correction, the number of true null hypotheses was estimated by using the histogram-based estimator for the obtained *p*-values [21]. Tests with q-values less than 0.2 were considered to be statistically significant.

Estimates of the regression coefficients obtained from the association analyses were scaled to units of SD of the resilience trait per SD of gene expression by dividing estimates by the SD of each trait. Also, signs of the estimates were reversed for phenotypes for which lower values are favorable, i.e. for TRT, MT, MOR, FCR, RFI, and back fat. The resulting adjusted estimates were used to compare results for traits that were measured in the same phase and for GSEA.

Gene set enrichment analyses were conducted using the GSEA_4.0.3 software [23], with gene sets of Gene Ontology biological processes (c5.bp.v7.1.symbols.gmt). Gene symbols were converted using human ortholog genes and biological processes with 10 or less genes in the data set or with 500 or more genes were filtered out, resulting in 9118 genes and 3824 terms remaining for analysis. The GSEA analyses were conducted separately for each analyzed phenotype using a gene list that was ranked by the adjusted regression coefficient estimates from the single gene association analyses, with the following options: number of permutations = 1000; collapse/remap to gene symbol = no_collapse; enrichment statistics = weighted. These analyses resulted in a normalized enrichment score and FDR for enrichment for each GO term and phenotype trait. GO terms that had FDR below chosen thresholds for at least one trait were then clustered based on their signed -log10(FDR) of enrichment for each trait, where the sign was based on whether an increase in expression of core genes in the GO term was associated with a favorable (+) or unfavorable (−) change in the trait. The resulting clusters and signed -log10(FDR) values were used to create heatmaps for the association of biological processes across resilience traits, using the pheatmap package in R [65].

## Supplementary Information

**Additional file 1: Fig. S1.** Illustration of gene annotation, combining the Ensembl (ENS) pig reference genome sequence assembly, release 92 (25,580 genes) and ISO-seq (ISO) based annotation (24,486 genes). **Fig. S2.** Distribution of genes based on the proportion of samples with non-zero count for each gene. Genes with non-zero counts in at least 80% of samples were used in further analyses (15,872 genes). **Fig. S3.** Boxplots of normalized read counts across the 912 samples by count per million (CPM; a) and the trimmed mean of M values (TMM; b) based on the EdgeR package in R (b). The log2 transformation was applied to the normalized counts plus 1 to obtain scaled expression values. **Fig. S4.** The number and overlap of genes whose expression was significantly (FDR < 0.10) affected by blood cell composition. There were no significant genes for basophile. **Fig. S5.** Relationship between associations of gene expression with (upper diagonal) or without (lower diagonal) adjustment for cell composition, with traits measured during the same phase, i.e. in the quarantine nursery (a), in the challenge nursery (b), in the finisher (c and d), across the challenge nursery and finisher (e), and at slaughter (f). Signs of the estimates were also reversed for resilience traits for which lower values are favorable (i.e. for treatment rate, mortality with treatments, mortality, feed conversion rate, residual feed intake, and back fat), such that a positive estimate always refers to a favorable change in the resilience trait associated with an increase in expression. The colors of blue, red, and orange indicate the significant associations (*q* < 0.20) for traits of x-axis, y-axis, and both traits, respectively. Correlation coefficients and its significance level (*p < 0.05, **p < 0.01, and ***p < 0.001) is shown in each scatter plot. **Fig. S6.** Illustrations for the criteria for adding 3’end extension for each transcript (a), and two types of the gene transfer format (GTF) files with the added 3’end window for use in QuantSeq-specific read count (b). The colors of blue, red, and green indicate the transcript of the gene A, the exons of the gene B, and the added 3′ end window, respectively. **Fig. S7.** Pipeline for the method to obtain QuantSeq-specific read counts, using an in-house Python script.

**Additional file 2.**

## Data Availability

The data were generated on commercially owned animals and, therefore, contains proprietary information. As a result, the data analysed in this study are not publicly available but are stored in a secure data base at the University of Alberta and they can be made available by the corresponding author on reasonable request.

## Abbreviations

RNA-seq: RNA-sequencing
QuantSeq: QuantSeq 3’mRNA sequencing
GB: Globin-blocker
WBC: white blood cell
eWO: mixed linear models without accounting for WBC composition for the adjustment of expression levels
eWI: mixed linear models with accounting for WBC composition for the adjustment of expression levels
SD: standard deviation
NDCM: the natural disease challenge model
qNUR: quarantine nursery
cNUR: challenge nursery
FIN: challenge finisher
HS: subjective health score
TRT: therapeutic treatment rate
MOR: mortality
M: million
ResWO: Residuals of the expression values that were obtained from the eWO models
ResWI: Residuals of the expression values that were obtained from the eWI models
FCR: feed conversion rate
GO: gene ontology
GSEA: gene set enrichment analysis
FDR: false discovery rate
RFI: residual feed intake
PRRSV: porcine reproductive and respiratory syndrome virus.
qNurHS1: health score on the day of entry into the quarantine nursery.
qNurHS2: health score on 2 weeks after entry into the quarantine nursery
cNurHS: health score on 2 weeks after entry into challenge nursery
cFinHS: health score on 6 weeks after entry into the challengefinisher
cNurTRT: number of therapeutic treatments per pig in challenge nursery
cFinTRT: number of therapeutic treatments per pig in challengefinisher
AllTRT: number of therapeutic treatments per pig in challenge nursery and finisher
cNurMOR: mortality rate for pigs in challenge nursery
cFinMOR: mortality rate for pigs in challengefinisher
AllMOR: mortality rate for pigs in challenge nursery and finisher
cNurMT: MT in challenge nursery
cFinMT: MT in challengefinisher
AllMT: MT across the challenge nursery and finisher
ADG: average daily gain
qNurADG: average daily gain in the quarantine nursery
cNurADG: average daily gain in challenge nursery
cFinADG: average daily gain in challenge finisher
ADFI: average daily feed intake
ADFD: average daily feed intake duration
RFI: residual feed intake
CWT: carcass weight
DRS: dressing percentage
LYLD: lean yield
CBF: carcass back fat
CLD: carcass lion depth
RIN: RNA integrity number
TMM: trimmed mean of M values
NES: normalized enrichment score

## References

[1] Guy SZ, Thomson PC, Hermesch S (2012). Selection of pigs for improved coping with health and environmental challenges: breeding for resistance or tolerance?. Front Genet.

[2] Doeschl-Wilson AB, Kyriazakis I (2012). Should we aim for genetic improvement in host resistance or tolerance to infectious pathogens?. Front Genet.

[3] Mulder HA, Rashidi H (2017). Selection on resilience improves disease resistance and tolerance to infections. J Anim Sci.

[4] Albers GA, Gray GD, Piper LR, Barker JS, Le Jambre LF, Barger IA (1987). The genetics of resistance and resilience to Haemonchus contortus infection in young merino sheep. Int J Parasitol.

[5] Wilkinson JM, Ladinig A, Bao H, Kommadath A, Stothard P, Lunney JK, Harding JC, Plastow GS (2016). Differences in whole blood gene expression associated with infection time-course and extent of fetal mortality in a reproductive model of type 2 porcine reproductive and respiratory syndrome virus (PRRSV) infection. PLoS One.

[6] Schroyen M, Eisley C, Koltes JE, Fritz-Waters E, Choi I, Plastow GS, Guan L, Stothard P, Bao H, Kommadath A, Reecy JM, Lunney JK, Rowland RR, Dekkers JC, Tuggle CK (2016). Bioinformatic analyses in early host response to porcine reproductive and respiratory syndrome virus (PRRSV) reveals pathway differences between pigs with alternate genotypes for a major host response QTL. BMC Genomics.

[7] Kommadath A, Bao H, Choi I, Reecy JM, Koltes JE, Fritz-Waters E, Eisley CJ, Grant JR, Rowland RR, Tuggle CK, Dekkers JC, Lunney JK, Guan LL, Stothard P, Plastow GS (2017). Genetic architecture of gene expression underlying variation in host response to porcine reproductive and respiratory syndrome virus infection. Sci Rep.

[8] Jaing C, Rowland RRR, Allen JE, Certoma A, Thissen JB, Bingham J, Rowe B, White JR, Wynne JW, Johnson D, Gaudreault NN, Williams DT (2017). Gene expression analysis of whole blood RNA from pigs infected with low and high pathogenic African swine fever viruses. Sci Rep.

[9] Lv J, Ding Y, Liu X, Pan L, Zhang Z, Zhou P, Zhang Y, Hu Y (2018). Gene expression analysis of porcine whole blood cells infected with foot-and-mouth disease virus using high-throughput sequencing technology. PLoS One.

[10] do Nascimento NC, Guimaraes AMS, Dos Santos AP, Chu Y, Marques LM, Messick JB: RNA-Seq based transcriptome of whole blood from immunocompetent pigs (*Sus scrofa*) experimentally infected with *Mycoplasma suis* strain Illinois. Vet Res. 2018, 49(1):49. 10.1186/s13567-018-0546-6 PMC6006945 29914581

[11] Lim KS, Dong Q, Moll P, Vitkovska J, Wiktorin G, Bannister S, Daujotyte D, Tuggle CK, Lunney JK, Plastow GS, Dekkers JCM (2019). The effects of a globin blocker on the resolution of 3'mRNA sequencing data in porcine blood. BMC Genomics.

[12] Moll P, Ante M, Seitz A, Reda T: QuantSeq 3′ mRNA sequencing for RNA quantification. Nat Methods. 2014, 11:i-iii.

[13] Shen-Orr SS, Gaujoux R (2013). Computational deconvolution: extracting cell type-specific information from heterogeneous samples. Curr Opin Immunol.

[14] Hwang B, Lee JH, Bang D (2018). Single-cell RNA sequencing technologies and bioinformatics pipelines. Exp Mol Med.

[15] Aran D, Hu Z, Butte AJ (2017). xCell: digitally portraying the tissue cellular heterogeneity landscape. Genome Biol.

[16] Monaco G, Lee B, Xu W, Mustafah S, Hwang YY, Carré C, Burdin N, Visan L, Ceccarelli M, Poidinger M, Zippelius A, Pedro de Magalhães J, Larbi A: RNA-Seq Signatures Normalized by mRNA Abundance Allow Absolute Deconvolution of Human Immune Cell Types. Cell Rep. 2019, 26(6):1627–1640.e7. 10.1016/j.celrep.2019.01.041 PMC6367568 30726743

[17] Whitney AR, Diehn M, Popper SJ, Alizadeh AA, Boldrick JC, Relman DA, Brown PO (2003). Individuality and variation in gene expression patterns in human blood. Proc Natl Acad Sci U S A.

[18] Putz AM, Harding JCS, Dyck MK, Fortin F, Plastow GS, Dekkers JCM; PigGen Canada Novel Resilience Phenotypes Using Feed Intake Data From a Natural Disease Challenge Model in Wean-to-Finish Pigs Front Genet 2019, 9:660. 10.3389/fgene.2018.00660 PMC6331689 30671080

[19] Cheng J, Putz AM, Harding JCS, Dyck MK, Fortin F, Plastow GS, Canada P, Dekkers JCM: Genetic analysis of disease resilience in wean-to-finish pigs from a natural disease challenge model. J Anim Sci. 2020, 98(8):skaa244. 10.1093/jas/skaa244 PMC7531181 32730570

[20] Beiki H, Liu H, Huang J, Manchanda N, Nonneman D, Smith TPL, Reecy JM, Tuggle CK (2019). Improved annotation of the domestic pig genome through integration of Iso-Seq and RNA-seq data. BMC Genomics.

[21] Nettleton D, Hwang JTG, Caldo RA, Wise RP (2006). Estimating the number of true null hypotheses from a histogram of P values. J Agr Biol Envir St.

[22] Van Gorp H, Van Breedam W, Delputte PL, Nauwynck HJ (2008). Sialoadhesin and CD163 join forces during entry of the porcine reproductive and respiratory syndrome virus. J Gen Virol.

[23] Subramanian A, Tamayo P, Mootha VK, Mukherjee S, Ebert BL, Gillette MA, Paulovich A, Pomeroy SL, Golub TR, Lander ES, Mesirov JP (2005). Gene set enrichment analysis: a knowledge-based approach for interpreting genome-wide expression profiles. Proc Natl Acad Sci U S A.

[24] Dong Q, Lunney JK, Lim KS, Nguyen Y, Hess AS, Beiki H, Rowland RRR, Walker K, Reecy JM, Tuggle CK, Dekkers JCM (2021). Gene expression in tonsils in swine following infection with porcine reproductive and respiratory syndrome virus. BMC Vet Res.

[25] Bai X, Putz AM, Wang Z, Fortin F, Harding JCS, Dyck MK, Dekkers JCM, Field CJ, Plastow GS, Canada P (2020). Exploring phenotypes for disease resilience in pigs using complete blood count data from a natural disease challenge model. Front Genet.

[26] Lester SN, Li K (2014). Toll-like receptors in antiviral innate immunity. J Mol Biol.

[27] Takeda K, Akira S (2004). TLR signaling pathways. Semin Immunol.

[28] Waddell LA, Lefevre L, Bush SJ, Raper A, Young R, Lisowski ZM, McCulloch MEB, Muriuki C, Sauter KA, Clark EL, Irvine KM, Pridans C, Hope JC, Hume DA (2018). ADGRE1 (EMR1, F4/80) is a rapidly-evolving gene expressed in mammalian monocyte-macrophages. Front Immunol.

[29] Ondrackova P, Leva L, Kucerova Z, Vicenova M, Mensikova M, Faldyna M (2013). Distribution of porcine monocytes in different lymphoid tissues and the lungs during experimental Actinobacillus pleuropneumoniae infection and the role of chemokines. Vet Res.

[30] Álvarez-Estrada Á, Rodríguez-Ferri EF, Martínez-Martínez S, Álvarez B, Fernández-Caballero T, Domínguez J, Gutiérrez-Martín CB (2019). TLR2, Siglec-3 and CD163 expressions on porcine peripheral blood monocytes are increased during sepsis caused by Haemophilus parasuis. Comp Immunol Microbiol Infect Dis.

[31] Fine DA, Rozenblatt-Rosen O, Padi M, Korkhin A, James RL, Adelmant G, Yoon R, Guo L, Berrios C, Zhang Y, Calderwood MA, Velmurgan S, Cheng J, Marto JA, Hill DE, Cusick ME, Vidal M, Florens L, Washburn MP, Litovchick L, DeCaprio JA (2012). Identification of FAM111A as an SV40 host range restriction and adenovirus helper factor. PLoS Pathog.

[32] He Y, Fan X, Liu N, Song Q, Kou J, Shi Y, Luo X, Dai Z, Yang Y, Wu Z, Wu G (2019). L-glutamine represses the unfolded protein response in the small intestine of weanling piglets. J Nutr.

[33] Gimsa U, Tuchscherer M, Kanitz E (2018). Psychosocial stress and immunity-what can we learn from pig studies?. Front Behav Neurosci.

[34] Kristiansen M, Graversen JH, Jacobsen C, Sonne O, Hoffman HJ, Law SK, Moestrup SK (2001). Identification of the haemoglobin scavenger receptor. Nature..

[35] Fairbairn L, Kapetanovic R, Sester DP, Hume DA (2011). The mononuclear phagocyte system of the pig as a model for understanding human innate immunity and disease. J Leukoc Biol.

[36] O'Connell GC, Tennant CS, Lucke-Wold N, Kabbani Y, Tarabishy AR, Chantler PD, Barr TL (2017). Monocyte-lymphocyte cross-communication via soluble CD163 directly links innate immune system activation and adaptive immune system suppression following ischemic stroke. Sci Rep.

[37] Nishino A, Katsumata Y, Kawasumi H, Hirahara S, Kawaguchi Y, Yamanaka H (2019). Usefulness of soluble CD163 as a biomarker for macrophage activation syndrome associated with systemic lupus erythematosus. Lupus..

[38] Fabriek BO, van Bruggen R, Deng DM, Ligtenberg AJ, Nazmi K, Schornagel K, Vloet RP, Dijkstra CD, van den Berg TK (2009). The macrophage scavenger receptor CD163 functions as an innate immune sensor for bacteria. Blood..

[39] Sánchez-Torres C, Gómez-Puertas P, Gómez-del-Moral M, Alonso F, Escribano JM, Ezquerra A, Domínguez J (2003). Expression of porcine CD163 on monocytes/macrophages correlates with permissiveness to African swine fever infection. Arch Virol.

[40] Whitworth KM, Rowland RR, Ewen CL, Trible BR, Kerrigan MA, Cino-Ozuna AG, Samuel MS, Lightner JE, McLaren DG, Mileham AJ, Wells KD, Prather RS (2016). Gene-edited pigs are protected from porcine reproductive and respiratory syndrome virus. Nat Biotechnol.

[41] Wells KD, Bardot R, Whitworth KM, Trible BR, Fang Y, Mileham A, Kerrigan MA, Samuel MS, Prather RS, Rowland RRR (2017). Replacement of porcine CD163 scavenger receptor cysteine-rich domain 5 with a CD163-like homolog confers resistance of pigs to genotype 1 but not genotype 2 porcine reproductive and respiratory syndrome virus. J Virol.

[42] Yang H, Zhang J, Zhang X, Shi J, Pan Y, Zhou R, Li G, Li Z, Cai G, Wu Z (2018). CD163 knockout pigs are fully resistant to highly pathogenic porcine reproductive and respiratory syndrome virus. Antivir Res.

[43] Dong Q: Genetics and transcriptomics of host response to PRRS in nursery pigs. 2019. Graduate Theses and Dissertations 17441. https://lib.dr.iastate.edu/etd/17441

[44] Boddicker N, Waide EH, Rowland RR, Lunney JK, Garrick DJ, Reecy JM, Dekkers JC (2012). Evidence for a major QTL associated with host response to porcine reproductive and respiratory syndrome virus challenge. J Anim Sci.

[45] Boddicker NJ, Bjorkquist A, Rowland RR, Lunney JK, Reecy JM, Dekkers JC (2014). Genome-wide association and genomic prediction for host response to porcine reproductive and respiratory syndrome virus infection. Genet Sel Evol.

[46] Koltes JE, Fritz-Waters E, Eisley CJ, Choi I, Bao H, Kommadath A, Serão NV, Boddicker NJ, Abrams SM, Schroyen M, Loyd H, Tuggle CK, Plastow GS, Guan L, Stothard P, Lunney JK, Liu P, Carpenter S, Rowland RR, Dekkers JC, Reecy JM (2015). Identification of a putative quantitative trait nucleotide in guanylate binding protein 5 for host response to PRRS virus infection. BMC Genomics.

[47] Ni L, Song C, Wu X, Zhao X, Wang X, Li B, Gan Y (2019). RNA-seq transcriptome profiling of porcine lung from two pig breeds in response to Mycoplasma hyopneumoniae infection. PeerJ..

[48] Truong AD, Hong YH, Lillehoj HS (2015). RNA-seq profiles of immune related genes in the spleen of necrotic enteritis-afflicted chicken lines. Asian-Australas J Anim Sci.

[49] Del Vesco AP, Kaiser MG, Monson MS, Zhou H, Lamont SJ (2020). Genetic responses of inbred chicken lines illustrate importance of eIF2 family and immune-related genes in resistance to Newcastle disease virus. Sci Rep.

[50] Sutherland MA, Backus BL, McGlone JJ (2014). Effects of transport at weaning on the behavior, physiology and performance of pigs. Animals (Basel).

[51] Cummins AG, Thompson FM (1997). Postnatal changes in mucosal immune response: a physiological perspective of breast feeding and weaning. Immunol Cell Biol.

[52] Bottje WG, Lassiter K, Piekarski-Welsher A, Dridi S, Reverter A, Hudson NJ, Kong BW (2017). Proteogenomics reveals enriched ribosome assembly and protein translation in Pectoralis major of high feed efficiency pedigree broiler males. Front Physiol.

[53] Lin YW, Wang J (2013). Structure and function of heme proteins in non-native states: a mini-review. J Inorg Biochem.

[54] Quintero-Gutiérrez AG, González-Rosendo G, Sánchez-Muñoz J, Polo-Pozo J, Rodríguez-Jerez JJ (2008). Bioavailability of heme iron in biscuit filling using piglets as an animal model for humans. Int J Biol Sci.

[55] Staroń R, Lipiński P, Lenartowicz M, Bednarz A, Gajowiak A, Smuda E, Krzeptowski W, Pieszka M, Korolonek T, Hamza I, Swinkels DW, Van Swelm RPL, Starzyński RR (2017). Dietary hemoglobin rescues young piglets from severe iron deficiency anemia: duodenal expression profile of genes involved in heme iron absorption. PLoS One.

[56] Ryu WS: Virus life cycle. Molecular Virology of Human Pathogenic Viruses 2017, 31–45.

[57] Gale M, Tan SL, Katze MG (2000). Translational control of viral gene expression in eukaryotes. Microbiol Mol Biol Rev.

[58] Park S, Yang JS, Shin YE, Park J, Jang SK, Kim S (2011). Protein localization as a principal feature of the etiology and comorbidity of genetic diseases. Mol Syst Biol.

[59] Harris N, Kunicka J, Kratz A (2005). The ADVIA 2120 hematology system: flow cytometry-based analysis of blood and body fluids in the routine hematology laboratory. Lab Hematol.

[60] Andrews SFASTQC (2010). A quality control tool for high throughput sequence data.

[61] Dobin A, Davis CA, Schlesinger F, Drenkow J, Zaleski C, Jha S, Batut P, Chaisson M, Gingeras TR (2013). STAR: ultrafast universal RNA-seq aligner. Bioinformatics..

[62] Anders S, Pyl PT, Huber W (2015). HTSeq--a Python framework to work with high-throughput sequencing data. Bioinformatics..

[63] Robinson MD, McCarthy DJ (2010). Smyth GK: edgeR: a bioconductor package for differential expression analysis of digital gene expression data. Bioinformatics..

[64] Bates D, Mächler M, Bolker B, Walker S (2016). Fitting linear mixed-effects models using lme4. J Stat Softw.

[65] Kolde R: Pheatmap: pretty heatmaps (R package version 1.0.12). 2019.

