# Supplementary material for: Quantitative analysis of the blood transcriptome of young healthy pigs and its relationship with subsequent disease resilience
Source: BMC Genomics. 2021 Aug 12;22:614. doi: 10.1186/s12864-021-07912-8 (PMC8361860; doi:10.1186/s12864-021-07912-8)
Supplement: Supplementary file 1 — Additional file 1: Fig. S1. Illustration of gene annotation, combining the Ensembl (ENS) pig reference genome sequence assembly, release 92 (25,580 genes) and ISO-seq (ISO) based annotation (24,486 genes). Fig. S2. Distribution of genes based on the proportion of samples with non-zero count for each gene. Genes with non-zero counts in at least 80% of samples were used in further analyses (15,872 genes). Fig. S3. Boxplots of normalized read counts across the 912 samples by count per million (CPM; a) and the trimmed mean of M values (TMM; b) based on the EdgeR package in R (b). The log2 transformation was applied to the normalized counts plus 1 to obtain scaled expression values. Fig. S4. The number and overlap of genes whose expression was significantly (FDR < 0.10) affected by blood cell composition. There were no significant genes for basophile. Fig. S5. Relationship between associations of gene expression with (upper diagonal) or without (lower diagonal) adjustment for cell composition, with traits measured during the same phase, i.e. in the quarantine nursery (a), in the challenge nursery (b), in the finisher (c and d), across the challenge nursery and finisher (e), and at slaughter (f). Signs of the estimates were also reversed for resilience traits for which lower values are favorable (i.e. for treatment rate, mortality with treatments, mortality, feed conversion rate, residual feed intake, and back fat), such that a positive estimate always refers to a favorable change in the resilience trait associated with an increase in expression. The colors of blue, red, and orange indicate the significant associations (q < 0.20) for traits of x-axis, y-axis, and both traits, respectively. Correlation coefficients and its significance level (*p < 0.05, **p < 0.01, and ***p < 0.001) is shown in each scatter plot. Fig. S6. Illustrations for the criteria for adding 3’end extension for each transcript (a), and two types of the gene transfer format (GTF) files with the added 3 [file 12864_2021_7912_MOESM1_ESM.pptx]

## Slide 1
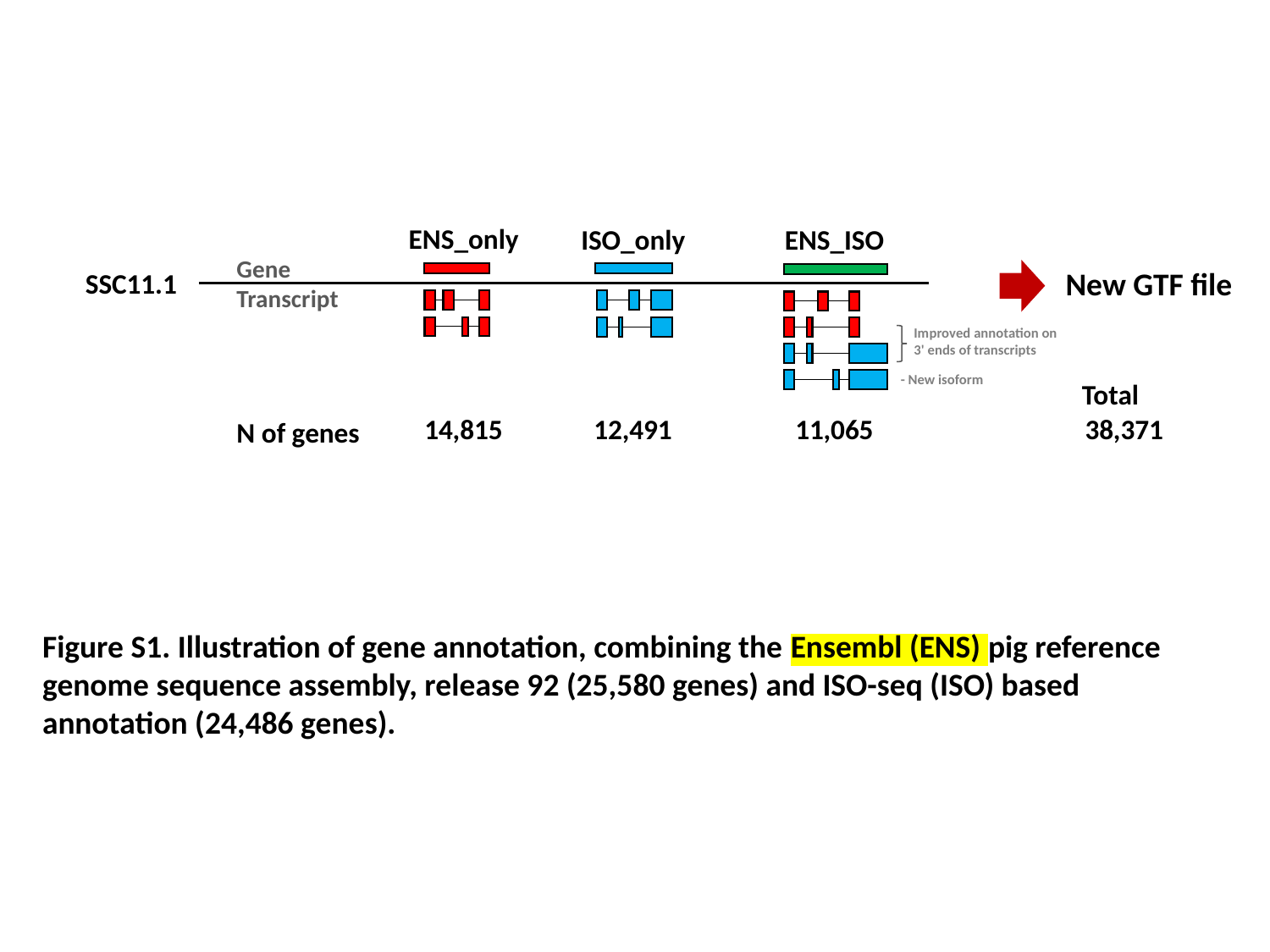

ENS_only
ISO_only
ENS_ISO
Gene
SSC11.1
Transcript
14,815
12,491
11,065
N of genes
New GTF file
Improved annotation on
3' ends of transcripts
- New isoform
Total
38,371
Figure S1. Illustration of gene annotation, combining the Ensembl (ENS) pig reference genome sequence assembly, release 92 (25,580 genes) and ISO-seq (ISO) based annotation (24,486 genes).

## Slide 2
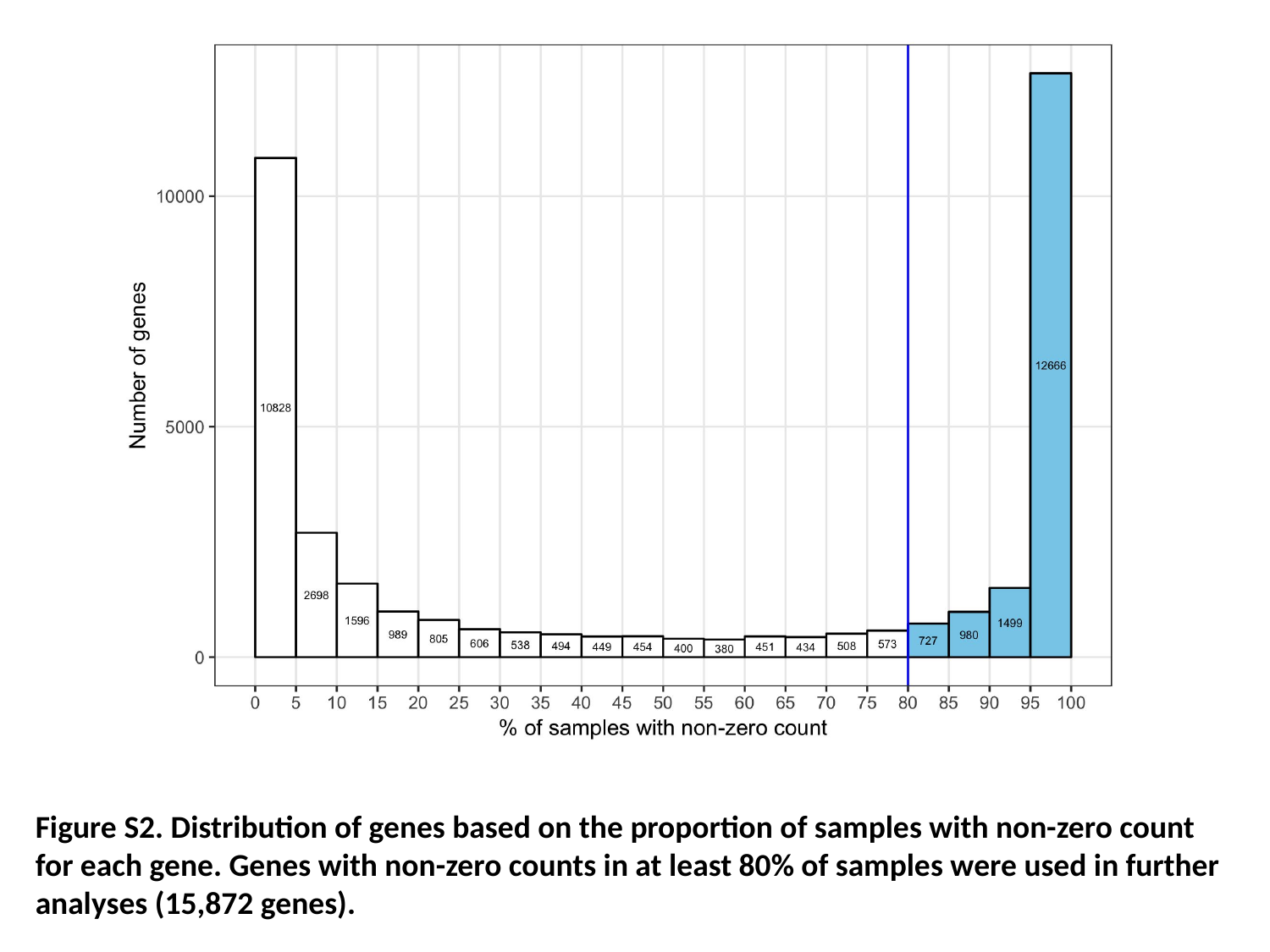

Figure S2. Distribution of genes based on the proportion of samples with non-zero count for each gene. Genes with non-zero counts in at least 80% of samples were used in further analyses (15,872 genes).

## Slide 3
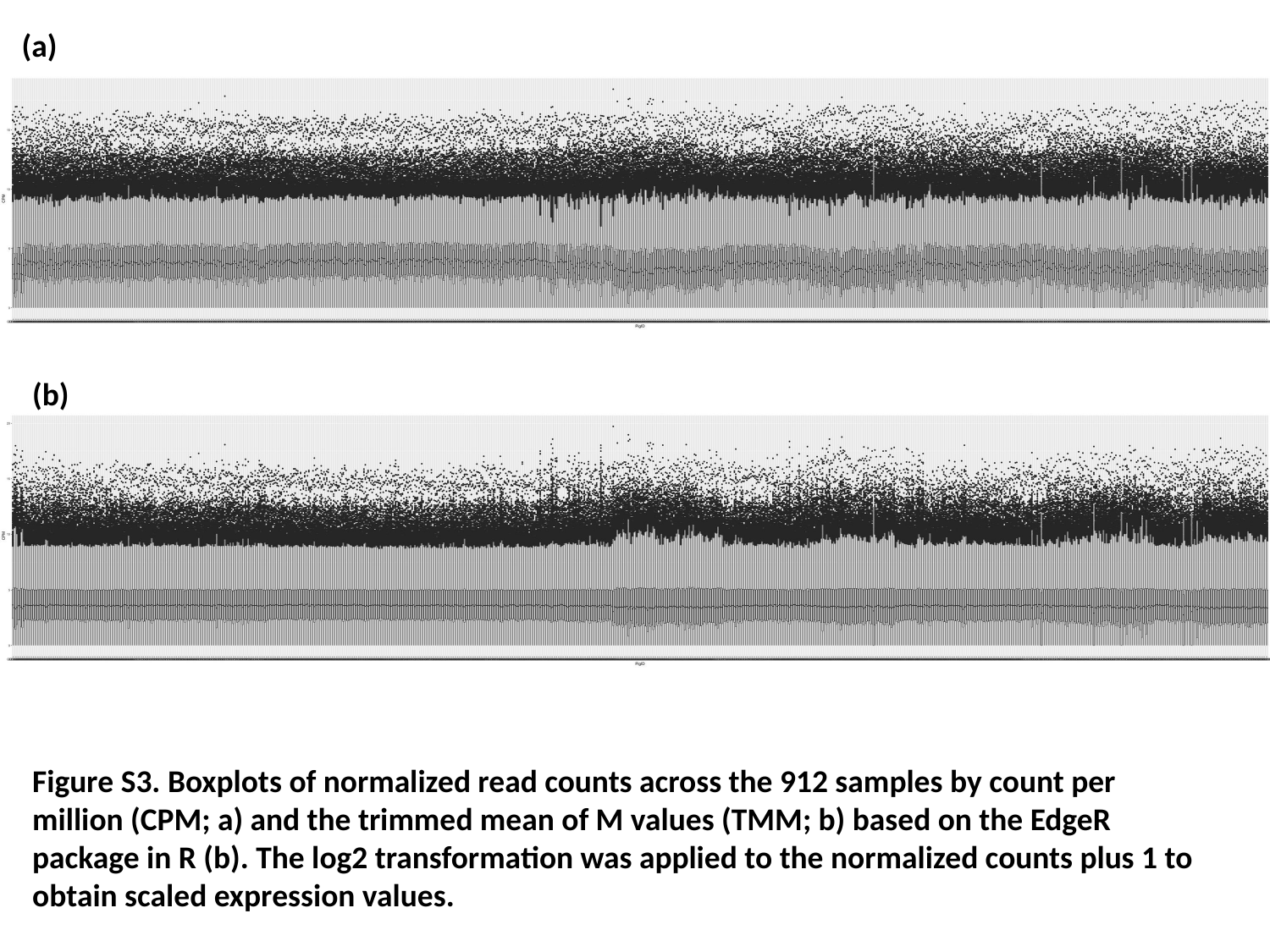

(a)
(b)
Figure S3. Boxplots of normalized read counts across the 912 samples by count per million (CPM; a) and the trimmed mean of M values (TMM; b) based on the EdgeR package in R (b). The log2 transformation was applied to the normalized counts plus 1 to obtain scaled expression values.

## Slide 4
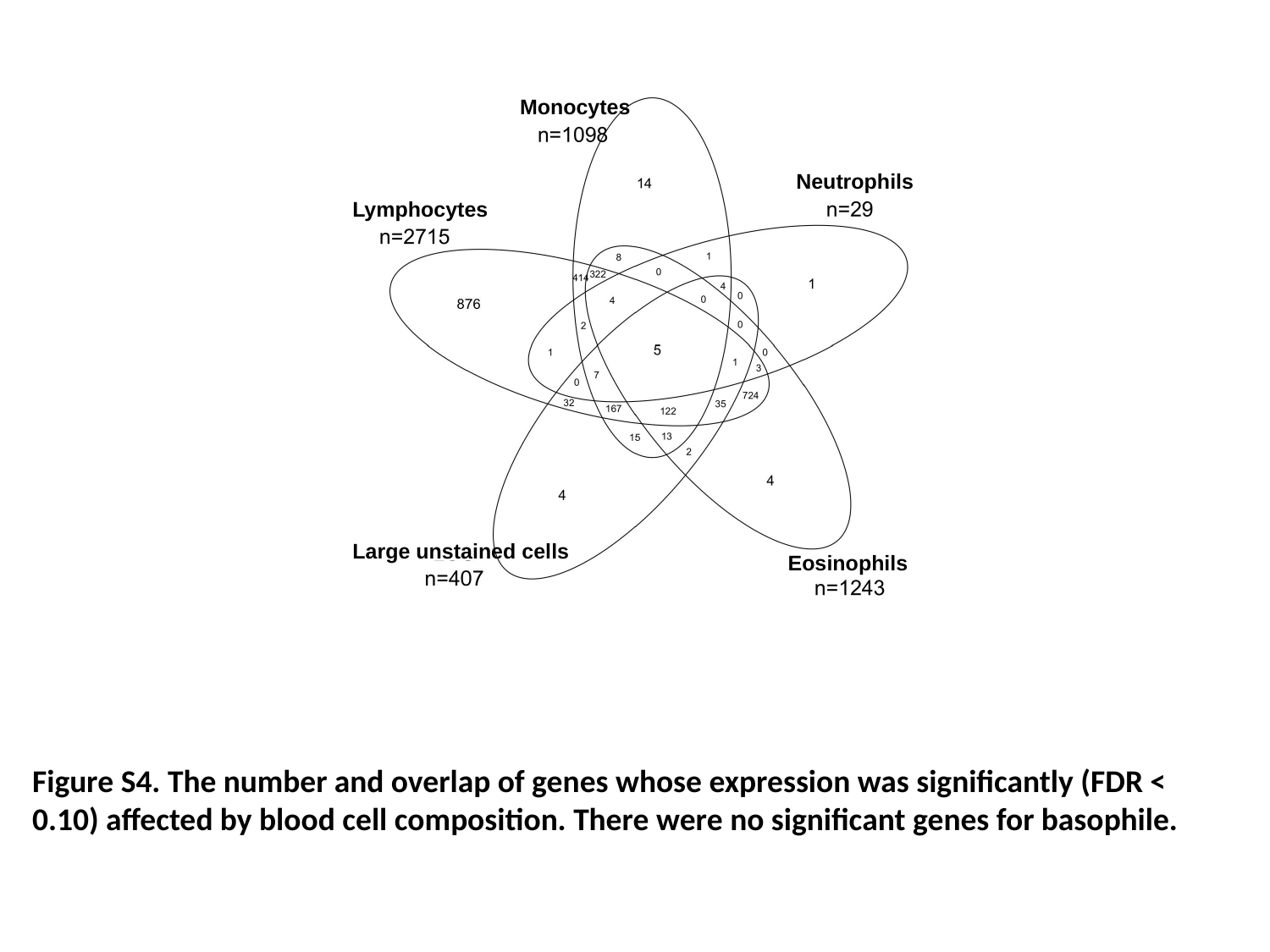

Monocytes
Neutrophils
Lymphocytes
Large unstained cells
Eosinophils
Figure S4. The number and overlap of genes whose expression was significantly (FDR < 0.10) affected by blood cell composition. There were no significant genes for basophile.

## Slide 5
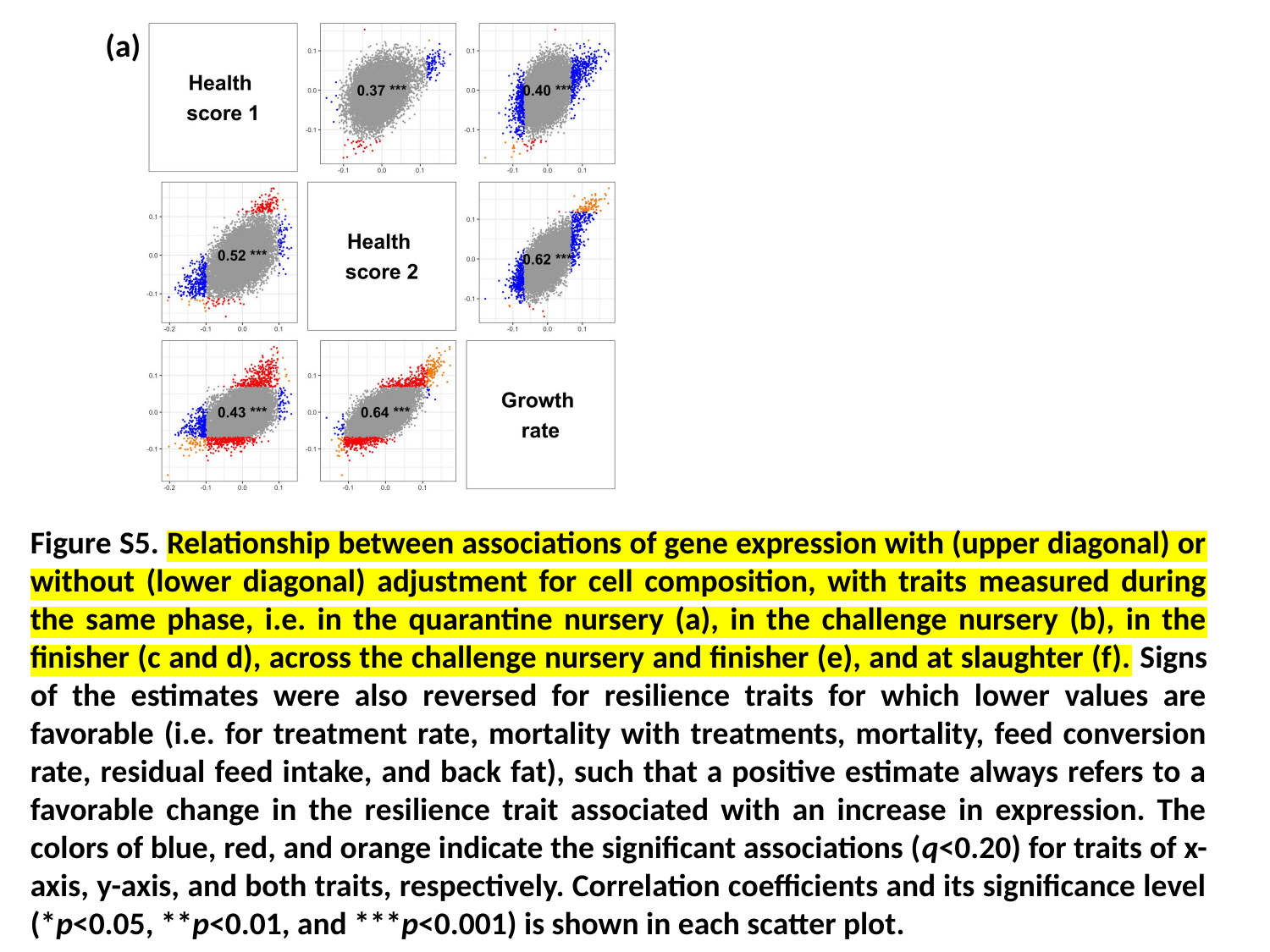

(a)
Figure S5. Relationship between associations of gene expression with (upper diagonal) or without (lower diagonal) adjustment for cell composition, with traits measured during the same phase, i.e. in the quarantine nursery (a), in the challenge nursery (b), in the finisher (c and d), across the challenge nursery and finisher (e), and at slaughter (f). Signs of the estimates were also reversed for resilience traits for which lower values are favorable (i.e. for treatment rate, mortality with treatments, mortality, feed conversion rate, residual feed intake, and back fat), such that a positive estimate always refers to a favorable change in the resilience trait associated with an increase in expression. The colors of blue, red, and orange indicate the significant associations (q<0.20) for traits of x-axis, y-axis, and both traits, respectively. Correlation coefficients and its significance level (*p<0.05, **p<0.01, and ***p<0.001) is shown in each scatter plot.

## Slide 6
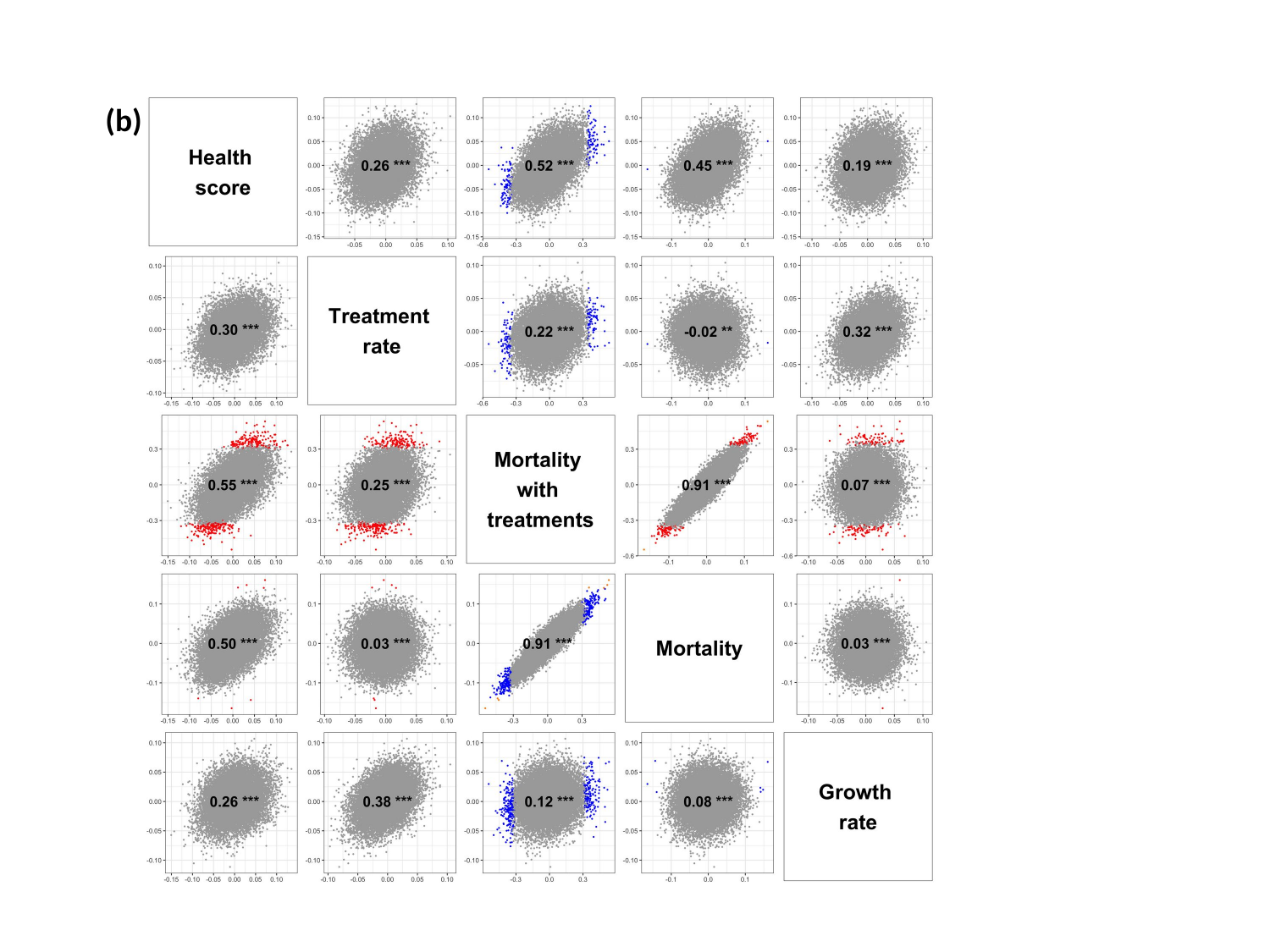

(b)

## Slide 7
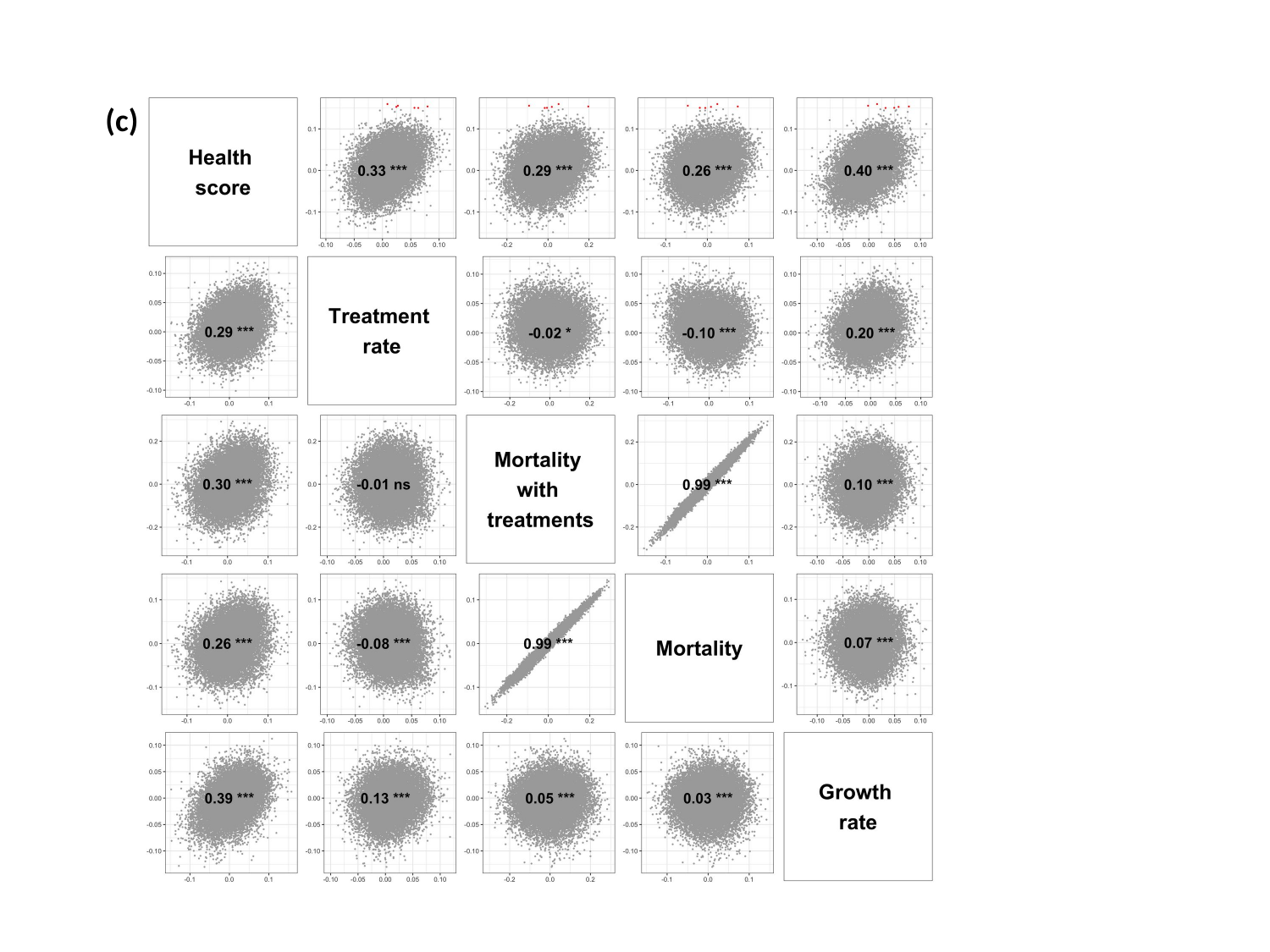

(c)

## Slide 8
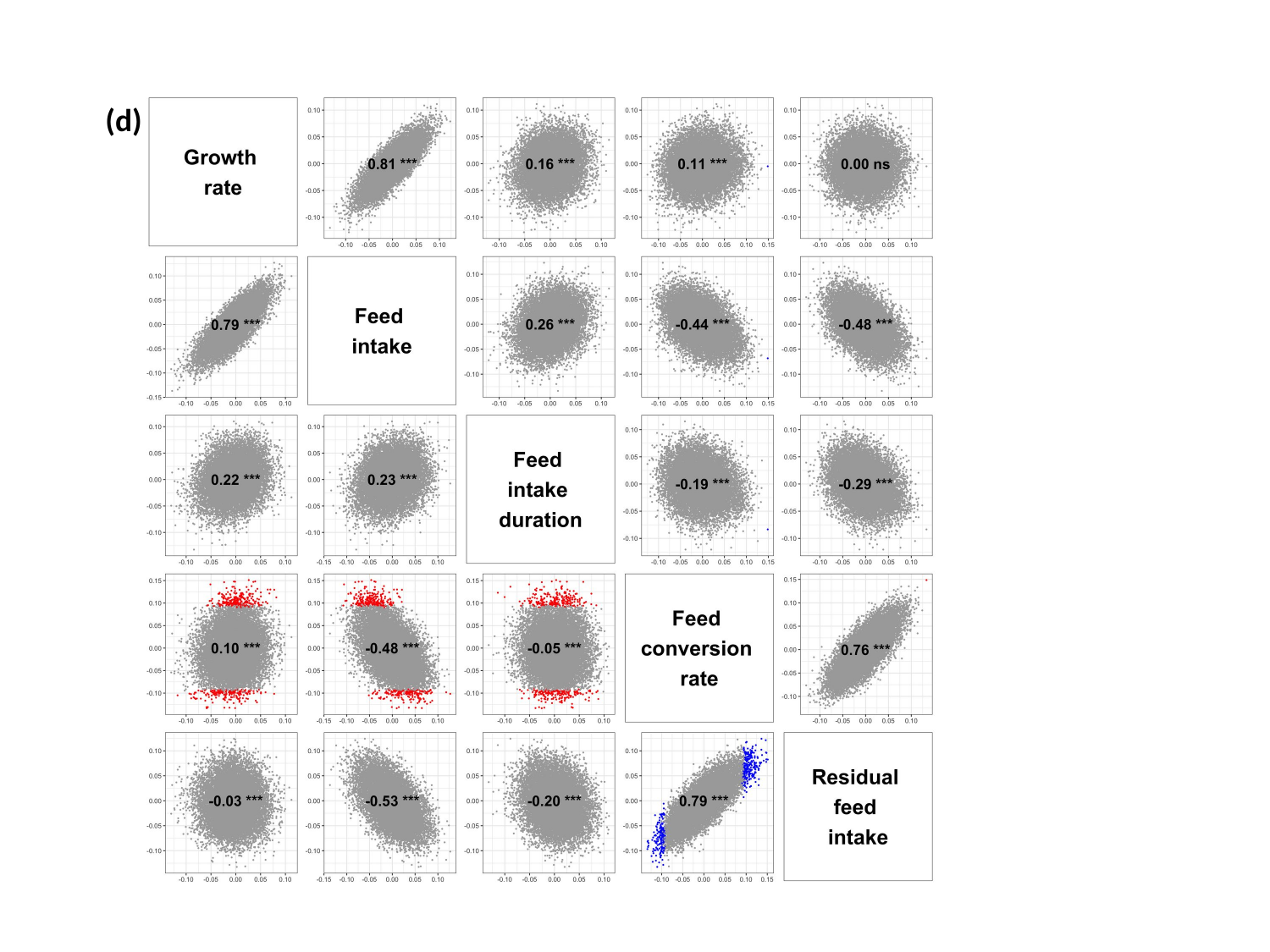

(d)

## Slide 9
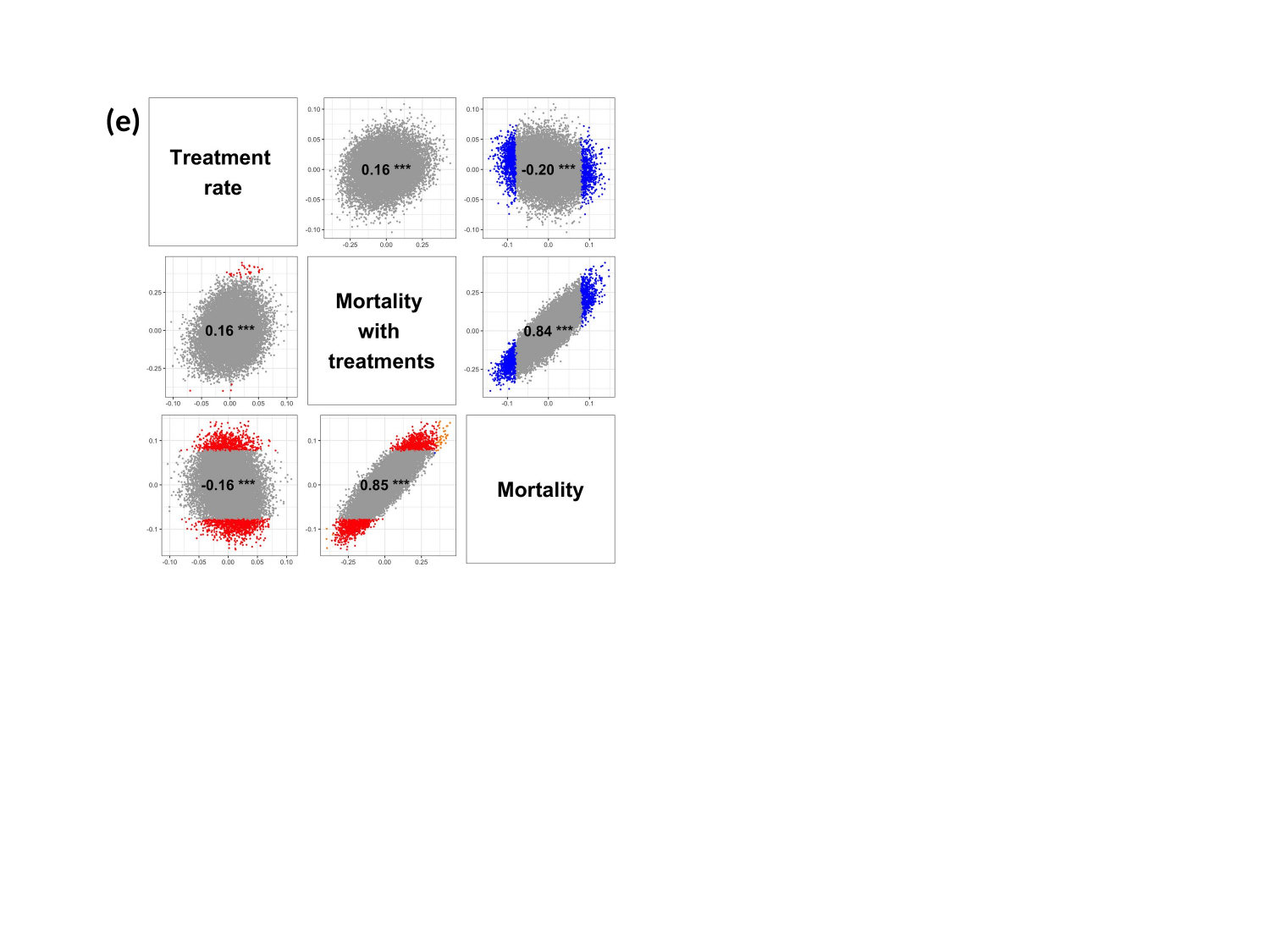

(e)

## Slide 10
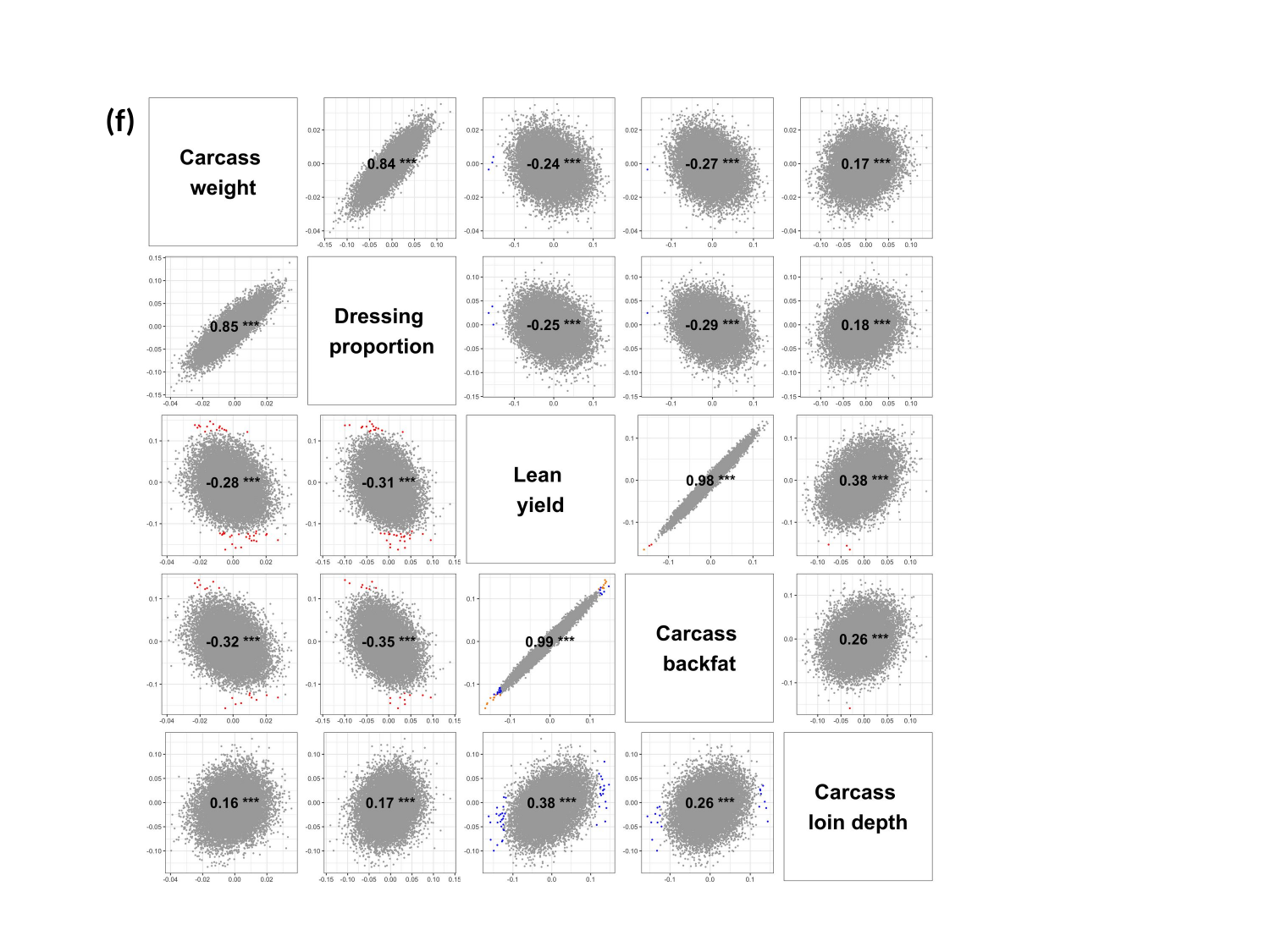

(f)

## Slide 11
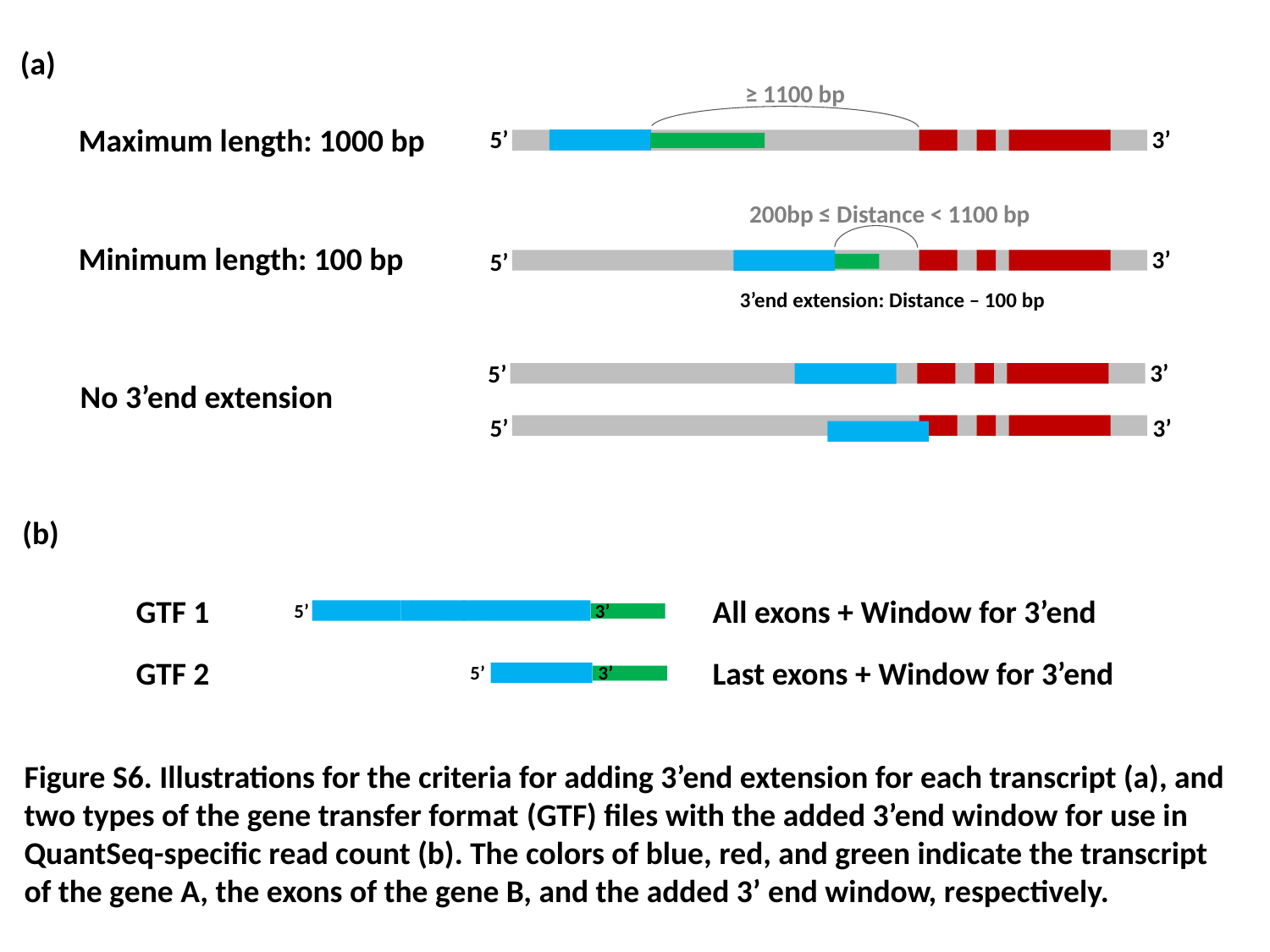

(a)
≥ 1100 bp
Maximum length: 1000 bp
3’
5’
200bp ≤ Distance < 1100 bp
Minimum length: 100 bp
3’
5’
3’end extension: Distance – 100 bp
3’
5’
No 3’end extension
3’
5’
(b)
GTF 1
All exons + Window for 3’end
5’
3’
GTF 2
Last exons + Window for 3’end
5’
3’
Figure S6. Illustrations for the criteria for adding 3’end extension for each transcript (a), and two types of the gene transfer format (GTF) files with the added 3’end window for use in QuantSeq-specific read count (b). The colors of blue, red, and green indicate the transcript of the gene A, the exons of the gene B, and the added 3’ end window, respectively.

## Slide 12
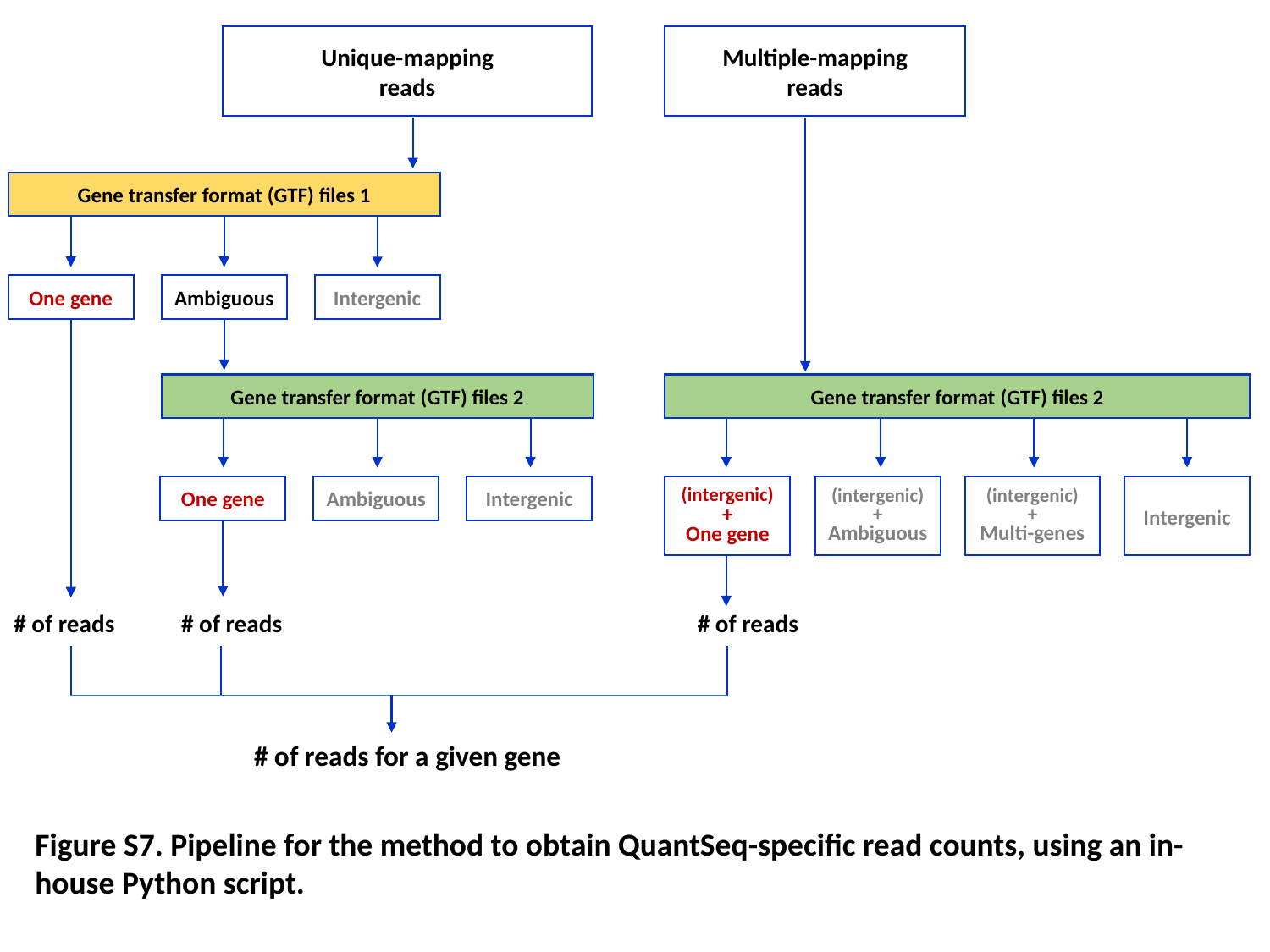

Unique-mapping
reads
Multiple-mapping
reads
Gene transfer format (GTF) files 1
One gene
Ambiguous
Intergenic
Gene transfer format (GTF) files 2
Gene transfer format (GTF) files 2
One gene
Ambiguous
Intergenic
(intergenic)+
One gene
(intergenic)+
Ambiguous
(intergenic)
+
Multi-genes
Intergenic
# of reads
# of reads
# of reads
# of reads for a given gene
Figure S7. Pipeline for the method to obtain QuantSeq-specific read counts, using an in-house Python script.
