# Supplementary material for: Quantitative analysis of the blood transcriptome of young healthy pigs and its relationship with subsequent disease resilience
Source: BMC Genomics. 2021 Aug 12;22:614. doi: 10.1186/s12864-021-07912-8 (PMC8361860; doi:10.1186/s12864-021-07912-8)
Supplement: Supplementary file 2 — Additional file 2. [file 12864_2021_7912_MOESM2_ESM.docx]

**Table S1.** Statistical models used for adjustment of gene expression data and for forward association analyses with resilience traits.

| Factor |  |  |  | Expression adjustment for CBC | |  | Trait category for association analysis ^a^ | | | |
| --- | --- | --- | --- | --- | --- | --- | --- | --- | --- | --- |
| Category | Factor Name | Type |  | Without | With |  | Quarantine nursery | Challenge nursery | Finisher | Carcass |
| Experimental batch | Batch | Fixed |  | V | V |  | V | V | V | V |
| Entry age to the facility | Entry Age | Covariate |  | V | V |  | V | V | V | V |
| Nursery enrichment | Enrichment | Fixed |  | - | - |  | V | V | V | V |
| Litter | Sow within batch | Random |  | - | - |  | V | V | V | V |
| Pen within batch | Quarantine nursery pen | Random |  | V | V |  | V | - | - | - |
|  | Challenge nursery pen | Random |  | - | - |  | - | V | - | - |
|  | Challenge finisher pen | Random |  | - | - |  | - | - | V | V |
| Slaughter | Date of slaughter | Fixed |  | - | - |  | - | - | - | V |
|  | Age of slaughter | Covariate |  | - | - |  | - | - | - | V |
|  | Pre-slaughter weight | Covariate |  | - | - |  | - | - | - | V |
| RNA quality | RNA integrity number | Covariate |  | V | V |  | - | - | - | - |
| Blood cell composition  (Log2 transformed proportion) | Lymphocytes | Covariate |  | - | V |  | - | - | - | - |
|  | Monocytes | Covariate |  | - | V |  | - | - | - | - |
|  | Neutrophils | Covariate |  | - | V |  | - | - | - | - |
|  | Eosinophils | Covariate |  | - | V |  | - | - | - | - |
|  | Basophils | Covariate |  | - | V |  | - | - | - | - |
|  | Large unstained cells | Covariate |  | - | V |  | - | - | - | - |
| Adjusted expression | Expression residual | Covariate |  | - | - |  | V | V | V | V |

^a^ Association tests for the binary variables such as health scores, mortality with treatments, mortality were conducted by reverse analysis which expression residual was response variable and the binary variable was fitted as fixed effect in the same model.

**Table S2**. The number of genes for which either ResWO or ResWI were more strongly associated with resilience phenotypes based on a likelihood ratio test (*p*<0.05).

| Trait measured  during each phase | Number of genes that fitted better | | |
| --- | --- | --- | --- |
|  | Without | With | Sum |
| ***Quarantine Nursery*** |  |  |  |
| Health score 1 | 24 | 25 | 49 |
| Health score 2 | 0 | 0 | 0 |
| Growth rate | 0 | 4 | 4 |
| ***Challenge Nursery*** |  |  |  |
| Health score | 0 | 0 | 0 |
| Treatment rate | 2 | 0 | 0 |
| Mortality + treatments | 1966 | 1850 | 3816 |
| Mortality | 0 | 0 | 0 |
| Growth rate | 1 | 0 | 1 |
| ***Challenge Finisher*** |  |  |  |
| Health score | 0 | 0 | 0 |
| Treatment rate | 0 | 0 | 0 |
| Mortality + treatments | 1032 | 1001 | 2033 |
| Mortality | 0 | 0 | 0 |
| Growth rate | 5 | 0 | 5 |
| Feed intake | 2 | 0 | 2 |
| Feed intake duration | 0 | 0 | 0 |
| Feed conversion rate | 185 | 8 | 193 |
| Residual feed intake | 0 | 2 | 2 |
| ***Overall Challenge*** |  |  |  |
| Treatment rate | 1 | 0 | 1 |
| Mortality + treatments | 1685 | 1513 | 3198 |
| Mortality | 0 | 0 | 0 |
| ***Carcass*** |  |  |  |
| Carcass weight | 0 | 3 | 3 |
| Dressing proportion | 2 | 4 | 6 |
| Lean yield | 17 | 10 | 27 |
| Carcass backfat | 15 | 6 | 21 |
| Carcass loin depth. | 0 | 0 | 0 |

**Table S3**. Estimates for the association of expression of the *CD163* gene with recorded phenotypes, with or without adjustment for cell composition (WBC).

| Trait measured by phase | Without WBC adjustment | |  | With WBC adjustment | |
| --- | --- | --- | --- | --- | --- |
|  | Estimate | *p*-value |  | Estimate | *p*-value |
| ***Quarantine Nursery*** |  |  |  |  |  |
| Health score 1 | -0.12 | <0.001^*^ |  | -0.10 | 0.003 |
| Health score 2 | -0.12 | 0.001^*^ |  | -0.07 | 0.06 |
| Growth rate | -0.10 | <0.001^*^ |  | -0.11 | <0.001^*^ |
| ***Challenge Nursery*** |  |  |  |  |  |
| Health score | -0.10 | 0.004 |  | -0.06 | 0.08 |
| Treatment rate | -0.06 | 0.04 |  | -0.03 | 0.31 |
| Mortality + treatments | -0.44 | <0.001^*^ |  | -0.42 | <0.001^*^ |
| Mortality | -0.12 | 0.001 |  | -0.12 | 0.001 |
| Growth rate | -0.05 | 0.05 |  | -0.03 | 0.28 |
| ***Challenge Finisher*** |  |  |  |  |  |
| Health score | -0.01 | 0.73 |  | -0.01 | 0.70 |
| Treatment rate | -0.06 | 0.05 |  | -0.06 | 0.05 |
| Mortality + treatments | -0.03 | 0.70 |  | -0.01 | 0.91 |
| Mortality | -0.01 | 0.80 |  | 0.00 | 0.95 |
| Growth rate | -0.03 | 0.37 |  | -0.05 | 0.19 |
| Feed intake | -0.04 | 0.29 |  | -0.02 | 0.56 |
| Feed intake duration | 0.01 | 0.83 |  | 0.00 | 0.91 |
| Feed conversion rate | 0.07 | 0.04 |  | 0.03 | 0.42 |
| Residual feed intake | 0.02 | 0.59 |  | -0.02 | 0.59 |
| ***Overall Challenge*** |  |  |  |  |  |
| Treatment rate | -0.07 | 0.02 |  | -0.05 | 0.11 |
| Mortality + treatments | -0.29 | 0.004 |  | -0.24 | 0.03 |
| Mortality | -0.08 | 0.02^*^ |  | -0.07 | 0.04 |
| ***Carcass*** |  |  |  |  |  |
| Carcass weight | 0.00 | 0.77 |  | 0.01 | 0.47 |
| Dressing proportion | 0.02 | 0.64 |  | 0.04 | 0.27 |
| Lean yield | 0.03 | 0.42 |  | 0.01 | 0.78 |
| Carcass backfat | 0.02 | 0.57 |  | 0.01 | 0.89 |
| Carcass loin depth | 0.03 | 0.33 |  | 0.02 | 0.61 |

^*^ Significant association (*q<*0.20)

**Table S4**. The effects of white blood cell types on the expression of the *CD163* gene in blood that was collected prior to disease challenge.

| Fixed effect | Estimate ^a^ | *q*-value ^a^ |
| --- | --- | --- |
| Lymphocytes | -1.3 | 5.92 x 10^-12^ |
| Monocytes | 0.62 | 2.30 x 10^-13^ |
| Neutrophils | 0.07 | 0.99 |
| Eosinophils | -0.1 | 0.14 |
| Basophile | 0.03 | 1 |
| Large unstained cells | 0.5 | 6.84 x 10^-06^ |

^a^ Estimates and significance levels from the mixed linear model with accounting for WBC composition.
